# Gut microbiome and fecal metabolite profiles in obese school-aged children from Northern Thailand

**DOI:** 10.3389/fmicb.2025.1657839

**Published:** 2025-09-11

**Authors:** Phatthanaphong Therdtatha, Lucsame Gruneck, Poramet Nachalam, Vasana Jinatham, Kritsakorn Saninjuk, Jiro Nakayama, Siam Popluechai

**Affiliations:** ^1^Specialized Research in Microbiome and Metabolome for Health Laboratory, Division of Biotechnology, Faculty of Agro-Industry, Chiang Mai University, Chiang Mai, Thailand; ^2^Gut Microbiome Research Group, Mae Fah Luang University, Chiang Rai, Thailand; ^3^Scientific and Technological Instruments Center, Mae Fah Luang University, Chiang Rai, Thailand; ^4^School of Science, Mae Fah Luang University, Chiang Rai, Thailand; ^5^Division of Applied Molecular Microbiology and Biomass Chemistry, Laboratory of Microbial Technology, Department of Bioscience and Biotechnology, Faculty of Agriculture, Graduate School, Kyushu University, Fukuoka, Japan

**Keywords:** gut microbiota, gut metabolites, BMI, obesity, school-aged children

## Abstract

Although the gut microbiota of school-aged children has been extensively studied, there is a significant lack of knowledge regarding the relationship between fecal metabolite profiles and the gut microbiota in the context of obesity in young children, particularly in Thailand. To elucidate this association, we analyzed fecal gut microbiota and metabolites of 67 school-aged children across various body mass index (BMI) and categorized: normal (*n* = 30), overweight (*n* = 20), and obese (*n* = 17), employing next-generation sequencing (NGS) and ultra-high-performance liquid chromatography coupled with quadrupole time-of-flight mass spectrometry (UHPLC-QTOF/MS), respectively. Obese children exhibited distinct profiles of both gut microbiota and metabolites compared to N and OW children. Genera enriched in the OB group included *Faecalibacterium, Collinsella, Megamonas, Brevundimonas,* and *Phascolarctobacterium*. Nearly 80 percent of distinct negative-ion features were more abundant in the higher BMI groups. Multivariate analyses revealed that BMI had a stronger influence on variations in fecal metabolite profiles than on gut microbiota composition. Shifts in association patterns between the gut microbiota and predicted microbial functions (KOs) were observed across BMI groups. Although no direct associations were observed between gut microbiota and metabolites, microbiome–metabolite interactions were predominantly mediated through microbial functions. Our findings highlight non-targeted metabolites associated with high BMI in school-aged children and illustrate microbiome–metabolite crosstalk through a microbe–function–metabolite triangle, which may be mediated through functional pathways rather than direct taxon–metabolite correlations.

## 1 Introduction

The human gastrointestinal (GI) tract, harboring a vast and diverse community of microbes known as the gut microbiome, has emerged as an important factor affecting the development of metabolic diseases (Wu et al., 2021). An imbalance in the composition of gut microbiome, known as gut dysbiosis, is the main factor driving metabolic diseases, primarily occurring in obesity (Bandopadhyay and Ganguly, 2022). Gut microbiome can affect the host metabolic alterations causing metabolic diseases by either directly or through its produced metabolites. For instance, short-chain fatty acids (SCFAs), produced by gut bacterial fermentation of carbohydrates, play a protective role against metabolic diseases by regulating energy and glucose homeostasis through G-protein-coupled receptors (Vogt et al., 2015; Priyadarshini et al., 2018). A reduction in SCFA-producing bacteria may contribute to gut dysbiosis and the development of metabolic disorders (Anachad et al., 2023). In contrast, branched-chain amino acids (BCAAs), which are synthesized by gut microbes, have been associated with insulin resistance and obesity, with dysregulated BCAA metabolism often preceding the onset of these conditions (Li et al., 2023). Currently, an increasing trend in studying gut metabolites to establish biomarker profiles in pathogenesis of metabolic diseases has been carried out using the metabolomic approaches (Jo et al., 2021; Calabrese et al., 2024). SCFAs, including butyrate, acetate, and propionate, are among the most extensively studied targeted metabolites in both obese children and adults (Li et al., 2025; Ecklu-Mensah et al., 2023; Kim et al., 2019). However, to the best of our knowledge, non-targeted metabolite profiling has yet to be explored in children with obesity, despite several reports in animal models and adults (Huazano-García et al., 2019; Rushing et al., 2021; Rushing et al., 2022). Non-targeted metabolite profiling in obese children is essential for identifying changed metabolic pathways, discovering new biomarkers, and understanding childhood obesity.

Childhood obesity is an escalating global health concern, particularly in Asia, where lifestyle changes have contributed to a rising prevalence of the condition (Therdtatha et al., 2022). Recent gut microbiome studies in Asian children have highlighted associations between microbial composition and body mass index (BMI) (Wang et al., 2024; Cho, 2021; Hou et al., 2017; Shin and Cho, 2020). In Thailand, research has demonstrated links between gut microbiota and childhood obesity. For example, children in Bangkok, who were more likely to be overweight, exhibited gut dysbiosis characterized by reduced levels of SCFA-producing bacteria compared to children in Buriram (Kisuse et al., 2018). Similarly, obese children in Bangkok and Pathum Thani showed decreased abundance of Actinobacteria (Visuthranukul et al., 2022; Burananat et al., 2025). Several bacterial taxa, including *Prevotella*, *Escherichia*, and *Clostridium*, have been positively associated with BMI. Although these previous studies mainly focused on gut microbiota composition, investigating fecal metabolite profiles using integrated multi-omics approaches, such as the combination of metabolomics and metagenomics, may provide a better understanding of the relationships between gut microbiota and obesity in children.

Existing studies on the gut microbiome of Thai children are limited, primarily focusing on the Central (Bangkok and Pathumthani) and Northeast (Buriram) regions. This overlooks the Northern region, particularly in terms of metabolomic studies across different BMI categories (Jitnarin et al., 2021), which may uniquely influence gut microbial composition and obesity. Additionally, regional dietary patterns in Northern Thailand could influence microbiome composition and metabolic profiles. Our previous study investigated the gut microbiome of Chiang Rai children associated with diet and demographic factors by using quantitative polymerase chain reaction (qPCR) (Gruneck et al., 2022). These findings revealed that obese children tended to consume a high-fat diet, which was associated with higher levels of *Gammaproteobacteria* and lower levels of Firmicutes (Bacillota) and *Ruminococcus*.

However, those findings were limited to the quantitative analysis of selected gut microbial targets and the lack of integration between metabolomics and microbiome. To gain deeper insight into the broader microbial community, particularly in the context of obesity, this current study further analyzed gut microbiome and non-targeted metabolite profiles using the same sample set from Gruneck et al. (2022). We employed next-generation sequencing (NGS) and ultra-high-performance liquid chromatography coupled with quadrupole time-of-flight mass spectrometry (UHPLC-QTOF/MS) to comprehensively profile gut microbiome and fecal metabolite profiles in obese children, compared to those with normal weight and overweight. This study is one of the first to integrate gut microbiota and non-targeted metabolomics in the obese school-aged children from Northern Thailand, an understudied population. Our multi-omics approach helps fill knowledge gaps by offering a comprehensive and unbiased view of the metabolic alterations associated with pediatric obesity.

## 2 Materials and methods

### 2.1 Ethic statement

The study was conducted according to the guidelines of the Declaration of Helsinki and was approved by the Ethics Committee of Mae Fah Luang University (EC23251-11) on 19 December 2023. The fecal samples analyzed in this study were obtained from a previous study (Gruneck et al., 2022), in which written informed consent was obtained from all participants. The current analysis used a subset of those previously collected and anonymized fecal samples, without involving any new participant contact or data collection.

### 2.2 Study participants and sample collection

This study included 67 school-aged children (6–15 years old), selected as a subset of 127 children from Ban Huai Rai Samakee Elementary School in Chiang Rai, Thailand (20 °16′13.6″N 99 °49′43.4″E). Details regarding participant recruitment and BMI classification have been previously described (Gruneck et al., 2022). Briefly, recruitment was conducted through voluntary participation coordinated by the school administration, with informed consent obtained from parents prior to participation. Inclusion criteria required children to be healthy, free from acute gastrointestinal diseases (e.g., gastroenteritis, diarrhea), and not to have taken antibiotics within 2 months before specimen collection. The number of participants was reduced from our previous study to balance group sizes and minimize confounding effects of ethnicity on gut microbiota composition by equalizing the number of individuals from each ethnic group (Gruneck et al., 2022). Participants were classified into normal weight (*N* = 30), overweight (OW = 20), and obese (OB = 17) groups based on gender-specific BMI-for-age z-scores, using World Health Organization cut-offs for children aged 5–19 years (De Onis et al., 2007). Fecal samples were collected on site in sterile containers, stored in ice boxes, and immediately transported to the laboratory for storage at −80 °C.

### 2.3 Extraction of fecal microbial DNA and amplicon sequencing of 16s rRNA genes

Fecal microbial DNA was extracted following the same protocol described in our previous study (Gruneck et al., 2022). For library preparation and NGS analysis, the extracted genomic DNA samples were submitted to Novogene Co., Ltd. (Singapore) and amplified using primers 341F-5′ CCTAYGGGRBGCASCAG 3′ and 806R-5′ GGACTACNNGGGTATCTAAT 3′, targeting V3-V4 variable regions of 16 s rRNA gene. Pooled DNA library was quantitatively assessed using Qubit® 2.0 (Life Technologies, CA, United States), before loading into Illumina Novaseq Xplus (Illumina, San Diego, CA, United States) paired-end reads 250 × 2 bp according to the standard protocols.

### 2.4 Bioinformatic analysis of amplicon sequence variants

Raw sequencing data was processed using Quantitative Insights Into Microbial Ecology 2 (QIIME2) pipeline version 2024.5.0 (Bolyen et al., 2019). Briefly, quality control was performed using the Cutadapt plugin (Martin, 2011) to remove adapter and primer sequences from both sequencing reads. Quality filtering, denoising, and merging were conducted using Divisive Amplicon Denoising Algorithm2 (DADA2) plugin (Callahan et al., 2016), generating amplicon sequence variants (ASVs) for phylogenetic and taxonomic analyses. Multiple sequence alignment of representative ASV sequences was performed using the MAFFT algorithm (Katoh and Standley, 2013). Phylogenetic tree was constructed using a maximum-likelihood method implemented in FastTree version 2.1.3 (Price et al., 2009). Taxonomic classification of ASVs was performed using the classify-sklearn Naive Bayes taxonomic classifier (Bokulich et al., 2018), with taxonomic assignment based on the Silva ribosomal RNA gene database (silva-138.2-ssu-nr99, released July 11, 2024).

### 2.5 Alpha and beta diversity analyses

Downstream analyses were conducted in R software (version 4.4.3) (R Core Team, 2025), with detailed summaries provided in Supplementary Data S1. Alpha and beta diversity were assessed at a rarefaction depth of 48,426 reads per sample, encompassing a total of 1,563 taxa (ASVs) (Supplementary File S1), using the *phyloseq* package (version 1.50.0) (McMurdie and Holmes, 2013). Alpha diversity metrics included Observed Species, Chao1 (species richness), Shannon (species richness and evenness), and PD Whole Tree (phylogenetic diversity). Group comparisons were performed using the Kruskal–Wallis test and visualized using *ggplot2* (version 3.5.2) (Wickham, 2016). Beta diversity was calculated using Unweighted UniFrac, Weighted UniFrac, and Bray–Curtis distance metrics. Principal Coordinate Analysis (PCoA) was performed using the ordinate function (*phyloseq*) and visualized with plot_ordination (based on *ggplot2*). Permutational Multivariate Analysis of Variance (PERMANOVA) based on the three distance metrics was conducted using the adonis2 function in the *vegan* package (version 2.6–10) (Oksanen et al., 2025), with 999 permutations. Pairwise PERMANOVA (permutation = 999) was then performed using the pairwise.adonis function in the *pairwiseAdonis* package (version 0.4.1). Homogeneity of group dispersions (permutation = 999) was tested using the betadisper function in *vegan*. Gut microbiota abundance was compared using the Kruskal–Wallis test (*p* < 0.05), followed by Dunn’s test with Benjamini–Hochberg (BH) correction (hereafter referred to as the *q*-value). The relative abundance of gut microbiota at the genus level was visualized using the *ComplexHeatmap* package (version 2.22.0) (Gu et al., 2016; Gu, 2022). Hierarchical clustering of rows and columns was performed based on Spearman correlation.

### 2.6 Metabolomic analysis of fecal samples

#### 2.6.1 Fecal sample preparation for metabolomic profiling

Due to limited remaining sample availability, only 55 fecal samples were subjected to metabolite analysis, comprising *N* = 24, OW = 18, and OB = 13. Approximately 0.6 g of fecal sample was mixed with ice-cold methanol at a 1:1 ratio (w/v) in a 2 mL Eppendorf tube and vortexed vigorously until homogenized. The homogenates were incubated at 4 °C overnight to allow for metabolite extraction, followed by centrifugation at 14,000 × *g* (12,000 rpm) for 10 min at 4 °C. The supernatants were filtered through a 0.22 μm syringe filter into new tubes and stored at −20 °C until UHPLC-QTOF/MS analysis.

#### 2.6.2 UHPLC QTOF/MS conditions

Non-targeted metabolomics profiling of fecal samples was conducted using an ultra-high-performance liquid chromatography coupled with quadrupole time-of-flight mass spectrometry (UHPLC-QTOF/MS) system (UHPLC 1290 Infinity II/6545B QTOF/MS system, Agilent, Santa Clara, United Stated States). Chromatographic separation was performed using an Agilent Poroshell 120 EC-C18 column (2.1 × 150 mm, 2.7 μm; Agilent, CA, United States) maintained at 35 °C. The mobile phases consisted of solvent A (0.1% formic acid in water) and solvent B (0.1% formic acid in acetonitrile), with a constant flow rate of 0.2 mL/min. The gradient elution program was as follows: 5–17% B from 0 to 13 min, 17–95% B from 13 to 25 min, and 95–5% B from 25 to 35 min (re-equilibration of the column to initial conditions). Detailed QTOF/MS instrument parameters are provided in Supplementary Data S2.

#### 2.6.3 Metabolite profile analysis

Raw data were processed using Agilent MassHunter Profinder (version 10.0, build 10.0.10142.1) to extract metabolite features based on the following criteria: peak height > 400 counts per second (cps), retention time (RT) tolerance ± 0.30 min, mass tolerance of 20 ppm, and an isotope model set to “organic molecule.” Feature extraction was conducted using batch recursive molecular feature extraction (BRMFE). The resulting features were subsequently analyzed using Agilent MassHunter Mass Profiler Professional (version 15.1, build 15.1.20045.0). Data alignment was performed using Pareto scaling. In total, 10,138 metabolites were detected across all fecal samples, comprising 6,469 features in the positive-ion mode and 3,669 in the negative-ion mode. Metabolites were filtered using one-way ANOVA (*p* < 0.05) and a fold-change threshold of >2.0. The filtered features were then subjected to partial least squares discriminant analysis (PLS-DA) to identify important features with variable importance in projection (VIP) scores greater than 1.0. Data outputs for each filtering step are provided in Supplementary File S2.

Non-targeted metabolites were mapped against the Kyoto Encyclopedia of Genes and Genomes (KEGG) Compound Database (https://www.genome.jp/kegg/compound/, accessed May 21, 2025) and the Human Metabolome Database (HMDB) (https://www.hmdb.ca/metabolites, accessed May 21, 2025) to retrieve compound descriptions and annotations. A total of 133 differential fecal metabolites across BMI groups with PLS-DA VIP scores > 1 (positive-ion mode = 95; negative-ion mode = 38) were selected for subsequent analyses. The metabolite abundance (log2) of both positive- and negative-ion mode metabolites was visualized using the *ComplexHeatmap* package (version 2.22.0). The heatmap represented log₂ differential metabolites with VIP > 1 (PLS-DA). Hierarchical clustering of rows and columns was performed based on Spearman correlation distance. The relative abundance (log₂ fold change) of both positive and negative ion mode metabolites (relative to the N group) was visualized using the *ggplot2* (version 3.5.2). Sparse Partial Least Squares Discriminant Analysis (sPLS-DA) was used to identify important metabolites that discriminate between BMI groups, implemented using the *mixOmics* package (version 6.30.0) (Rohart et al., 2017). Log₂ values were scaled prior to modeling. Component and variable selection were optimized by tuning the model with 5-fold cross-validation repeated 100 times. Model performance was evaluated using Receiver Operating Characteristic (ROC) curves and the Area Under the Curve (AUC). The classification accuracy of sPLS-DA models was interpreted as follows: no discrimination (AUC = 0.5), low (AUC = 0.6–0.7), acceptable (AUC = 0.7–0.8), excellent (AUC = 0.8–0.9), and outstanding (AUC > 0.9) (Lê et al., 2008; Kassambara and Mundt, 2020). Features with the loading values (VIP > 1) contributing maximally to group separation on each component were visualized *ggplot2* (version 3.5.2).

### 2.7 Multivariate statistical analysis of associations between gut microbiota and metabolite profiles

Differentially abundant gut microbiota at the genus level (26 variables, CLR-transformed), positive-ion metabolites (95 variables, log₂-transformed), and negative-ion metabolites (38 variables, log₂-transformed) across BMI groups were integrated using Multiple Factor Analysis (MFA) to identify key features contributing to overall variation. MFA was conducted using the *FactoMineR* package (version 2.11) (Lê et al., 2008), and the *factoextra* package (version 1.0.7) (Kassambara and Mundt, 2020) was used to visualize variable contributions and correlations in the MFA space. To further investigate the influence of BMI groups on variation in gut microbiota (144 genera) and metabolite profiles (positive-ion mode = 95; negative-ion mode = 38), as well as the relationships between them, redundancy analysis (RDA) was performed using the *vegan* package. The BMI group was treated as a constrained explanatory variable, while the gut microbiota (CLR-transformed) and metabolite data (log₂-transformed and scaled) were used as response variables. The significance of the constraints was assessed using an ANOVA-like permutation test (999 permutations). RDA biplots were generated with type 1 scaling (distances between samples represent similarity) and type 2 scaling (angles between arrows reflect correlations among variables), and visualized using *ggplot2*.

### 2.8 Functional profile analysis of gut microbiota

The functional profiles of gut microbiota were predicted using PICRUSt2 (version 2.6.2) based on KEGG Orthology (KO). Annotated genes were represented as K numbers and mapped against the KEGG database (http://rest.kegg.jp/get/br:ko00001, accessed May 30, 2025) to obtain pathway-level functional annotations. Differentially abundant KOs across BMI groups were identified using the ALDEx2 package (version 1.38.0). Welch’s *t*-test (*we.ep*) and Welch’s *t*-test Benjamini-Hochberg adjusted *q*-values (*we.eBH*) were used to determine significance. Pairwise comparisons included N vs. OW, N vs. OB, and OB vs. OW. Associations were evaluated between gut microbiota (144 genera) and 5,819 predicted KOs, between fecal metabolites (positive-ion mode: 95; negative-ion mode: 38) and 5,819 predicted KOs, and between fecal metabolites and KO abundance stratified by contributing ASVs at the genus level (KO|Genus) based on significantly differentially abundant KOs identified via ALDEx2. All associations were assessed using the Hierarchical All-against-All Association (HAllA) method (Ghazi et al., 2022), implemented in Python (version 3.10.16). Significant associations (Spearman correlation coefficient *r* ≥ *|*0.7*|*, *q* < 0.05) were further analyzed through network analysis using the *igraph* package (version 2.1.4) (Csárdi et al., 2025). Community detection in the network was performed using the Louvain clustering algorithm.

## 3 Results

### 3.1 Gut microbiota diversity across BMI groups

Microbial diversity was assessed at a sequence depth of 48,426 reads/sample, retaining 1,563 ASVs. Alpha diversity analysis showed no significant difference among BMI groups (Supplementary Figure S1) based on four indices including observed species, Chao1, Shannon, and PD. Beta diversity based on the unweighted UniFrac (*F*_paired. PERM_ = 1.95, *q* = 0.006) revealed significant differences in community membership between N and OB groups, with no significant difference in dispersion (Supplementary Figure S2). No significant differences in overall community structure and composition were observed using the weighted UniFrac and Bray-Curtis distance matrices.

### 3.2 Compositional differences in gut microbiota across BMI groups

Based on 1,563 ASVs conserved across all samples, 11 known ASVs were identified at the phylum level, 17 at class, 30 at order, 48 at family, and 143 at genus level. ASVs that could not be classified from class to genus were grouped as unclassified. Microbiome composition analysis revealed that the five most abundant phyla across all BMI groups were Bacteroidota, Bacillota, Actinomycetota, Verrucomicrobiota, and Fusobacteriota (Supplementary Figure S3A). A significant difference was observed for Actinomycetota (*q* = 0.02), Verrucomicrobiota (*q* = 0.02) and Elusimicrobiota (*q* = 0.03) between N and OB groups; however, the latter phylum was present in only one sample within the OB group (Supplementary Figure S3B; Supplementary File S3).

At the class level, three bacterial taxa showed significant differences between BMI groups (Supplementary Figure S4A). *Coriobacteriia* and *Negativicutes* were significantly more abundant in the OB group compared to both N and OW groups (*q* < 0.05), while *Elusimicrobia* was also more abundant in OB compared to N (*q* = 0.023). Other dominant classes such as *Bacteroidia*, *Clostridia*, *Verrucomicrobiia*, *Actinobacteria*, *Bacilli*, *Fusobacteriia*, and *Alphaproteobacteria* did not show significant differences among BMI groups.

At the order level, *Coriobacteriales*, *Veillonellales*-*Selenomonadales*, *Elusimicrobiales*, *Acidaminococcales*, and *Caulobacterales* showed the highest abundance in the OB group compared to the N and OW groups (*q* < 0.05) (Supplementary Figure S4B). At the family level, *Rikenellaceae* had the lowest abundance in the OB group compared to N and OW (*q* < 0.05) (Supplementary Figure S5). *Barnesiellaceae* and *Marinifilaceae* were more abundant in the N group than in OB (*q* < 0.05), whereas *Coriobacteriaceae*, *Selenomonadaceae*, *Acidaminococcaceae*, and *Caulobacteraceae* were most enriched in the OB group relative to the N and OW groups (*q* < 0.05). *Elusimicrobiaceae* abundance was significantly higher in OB than in N (*q* = 0.008).

**Figure 1:**
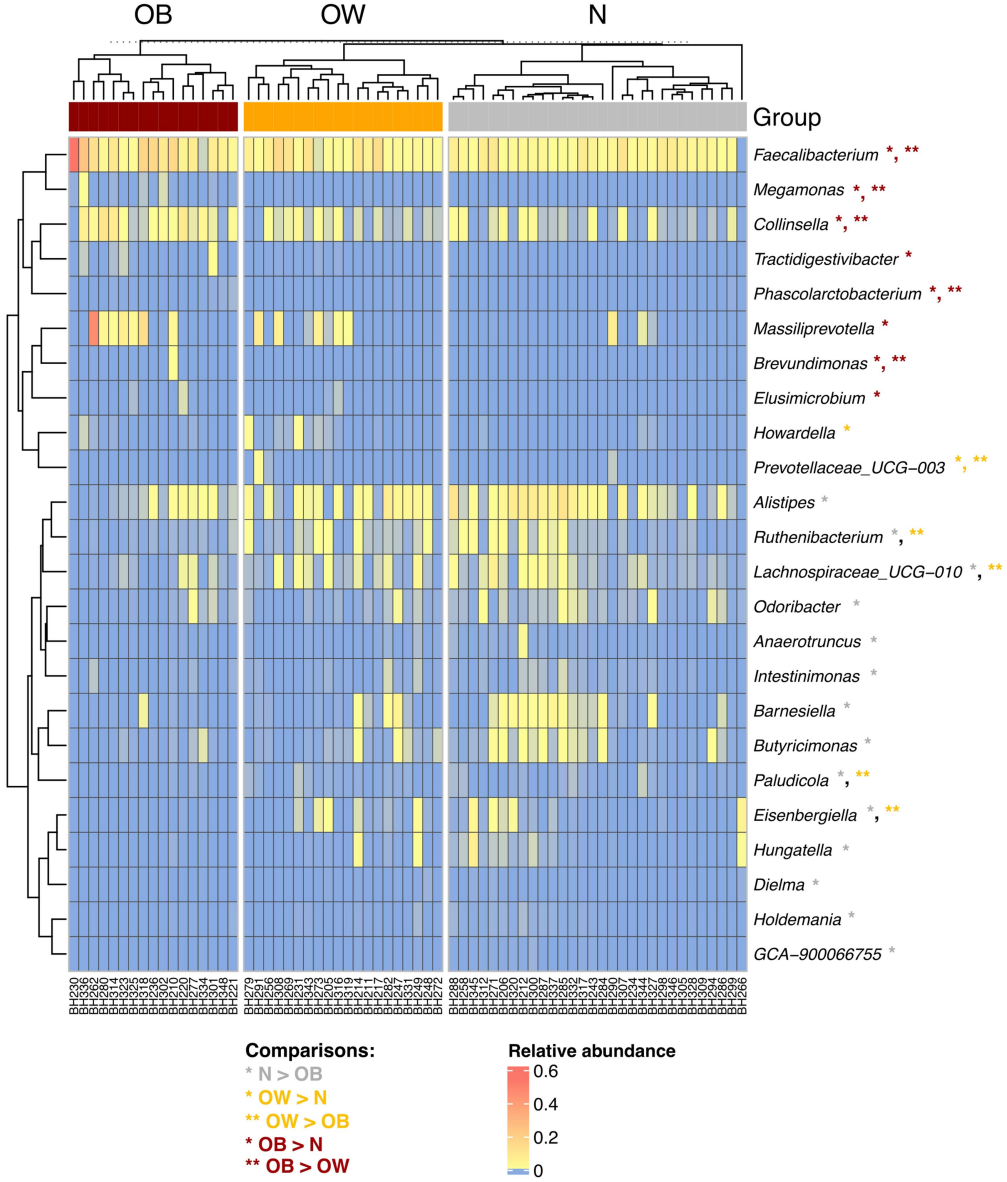
Hierarchical clustering of 24 significantly different genera between BMI groups. The heatmap represents the relative abundance of gut microbiota at the genus level. Abundance levels range from high (red) to low (blue). Asterisks (*) indicate genera with significant differences between groups, identified using *post hoc* Dunn’s test with Benjamini-Hochberg correction (*q* < 0.05), following a significant Kruskal-Wallis test (*p* < 0.05). Rows and columns were clustered based on Spearman correlation distance. BMI groups: *N* = normal weight (*n* = 30); OW = overweight (*n* = 20); OB = obese (*n* = 17).

At the genus level, *Segatella* and *Bacteroides* were the most prevalent genera across all groups (Supplementary Figure S6). Among the 24 genera with significant differences between BMI groups, five were most prevalent in the OB groups (*q* < 0.05), including *Faecalibacterium*, *Collinsella*, *Megamonas*, *Brevundimonas*, and *Phascolarctobacterium* (Figure 1). Additionally, *Massiliprevotella*, *Tractidigestivibacter*, and *Elusimicrobium* were also more abundant in OB compared to N (*q* < 0.05). *Prevotellaceae UCG-003* was most enriched in the OW group, while the abundance of *Howardella* was also higher in OW compared to N (*q* < 0.05). In contrast, *Lachnospiraceae UCG-010*, *Eisenbergiella*, *Ruthenibacterium*, and *Paludicola* exhibited lowest abundance in the OB group (*q* < 0.05). The abundance of 10 genera, including *Alistipes*, *Barnesiella*, *Hungatella*, *Butyricimonas*, *Odoribacter*, *Intestinimonas*, *Anaerotruncus*, *Holdemania*, *Dielma*, and *GCA-900066755*, were also significantly less abundant in OB compared to N (*q* < 0.05). Overall, the gut microbiota composition in OB participants was more distinct from that of N than OW, highlighting obesity-associated changes in microbial profiles.

### 3.3 Distinct fecal metabolite profiles between N and OB groups

**Figure 2:**
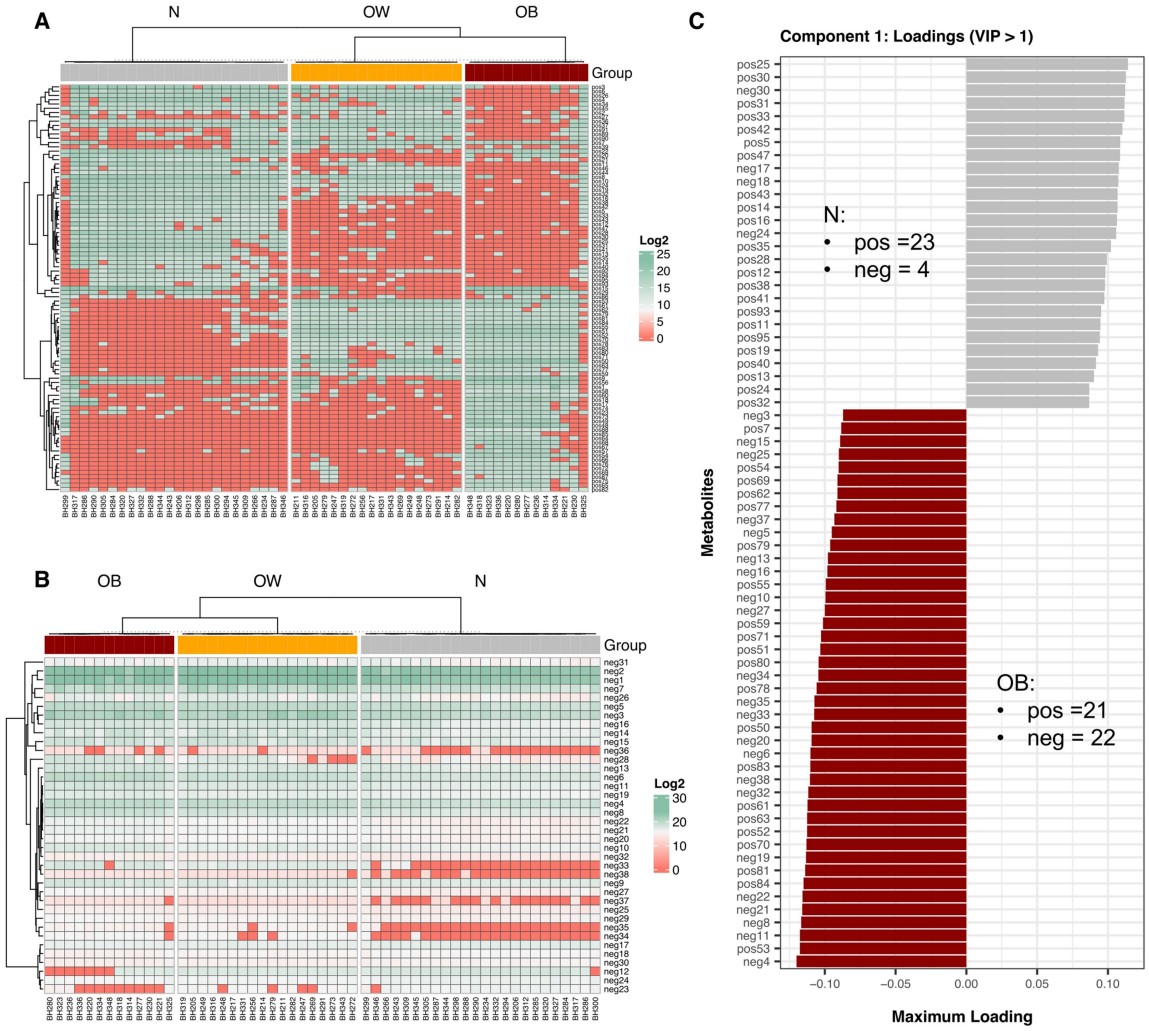
Fecal metabolite abundance across BMI groups. Heatmap showing hierarchical clustering of differential metabolites detected in positive-ion mode **(A)** and negative-ion mode **(B)**, with PLS-DA VIP scores > 1. Metabolite abundance is displayed as Log₂ values, with green indicating higher abundance and red indicating lower abundance. Both rows (metabolites) and columns (samples) were clustered using Spearman correlation distance. **(C)** Bar plots display discriminant metabolites from the tuned sPLS-DA model (VIP > 1) with the highest loading scores along component 1, based on maximum (positive) and minimum (negative) group mean coordinates. Horizontal bars represent individual metabolites (positive- or negative-ion modes) contributing to group separation, with bar length proportional to the loading weight. BMI groups: N = normal weight (*n* = 24); OW = overweight (*n* = 18); OB = obese (*n* = 13). pos = positive-ion mode; neg = negative-ion mode.

Using a PLS-DA cutoff of VIP > 1, a total of 133 differential fecal metabolites were identified across BMI groups (Figures 2A,B), of which 95 were detected in positive-ion mode and 38 in negative-ion mode. The mean log₂ fold change (log₂FC) of positive-ion metabolites relative to the N group revealed that 22 positive-ion features were upregulated, 70 were downregulated, and 3 remained unchanged in the OW group (Supplementary Figure S7; Supplementary File S4). In the obese (OB) group, 40 positive-ion features were upregulated, while 55 were downregulated. For negative-ion metabolites, 31 features were consistently upregulated in both OW and OB groups, whereas only 7 features were downregulated in these two groups compared to the N group. Additionally, direct comparisons of log₂-transformed positive-ion metabolite abundances revealed that 27 features were most enriched in the OB group (*q* < 0.05), whereas 30 and 4 features were most abundant in the N and OW groups, respectively (*q* < 0.05; Supplementary File S4). For negative-ion metabolites, 30 out of 38 features were least abundant in the N group compared to both OW and OB groups (*q* < 0.05), suggesting an increased abundance of these metabolites in individuals with higher BMI. Notably, the OW group exhibited a negative metabolite profile more similar to that of the OB group than to the N group. Nearly half of these negative-ion metabolites (16 features) were significantly enriched in the OB group (*q* < 0.05), further emphasizing their association with higher BMI.

Further sPLS-DA analysis based on a tuned model (VIP score > 1) identified 70 and 23 metabolite features (across both ion modes) as important for discriminating between BMI groups in components 1 and 2, respectively (Supplementary File S4). Component 1, which explained 53% of the variance, revealed fewer positive-ion metabolites associated with the OB group (21, AUC = 96, *p* < 0.0001) compared to the N group (23, AUC = 97, *p* < 0.0001), while 22 negative-ion metabolites contributed to the separation of OB from N (Figure 2C). The top five metabolites enriched in OB were neg4 (Ala Lys Pro Gln), pos53 (unclassified), neg11 (JWH 018 N-(5-hydroxypentyl) metabolite-d5), neg8 (Sarcostin), and neg 21 (15-keto-Prostaglandin E2). Only four negative metabolites contributed to the N group. In component 2, which accounted for for 14% of explained variance, more positive-ion compounds were associated with OB (16, AUC = 0.94, *p* < 0.0001) than with OW (7, AUC = 1, *p* < 0.0001) (Supplementary Figure S8B). The top contributors for OB included pos20 (L-Phenylalanyl-L-valyl-L-leucyl-L-prolyl-L-tryptophyl-N ~ 5 ~ −(diaminomethylidene)-L-ornithyl-L-isoleucine), pos88 (Amaranthussaponin II), pos65 (PC(16:0/9:0(CHO))), pos48 (unclassified), and pos23 (unclassified), while pos37 (unclassified) and pos91 [*Endothall, di(N, N-dimethyltridecylamine) salt (1:2)*] were highly associated with OW. Among these, pos88 (Amaranthussaponin II; C₄₈H₇₄O₂₀; HMDB0041352) is an organic compound classified as a lipid-like molecules and functions as an energy source; however, no specific KEGG pathway annotation was identified.

### 3.4 BMI groups more strongly influenced variations in fecal metabolite profiles than gut microbiota composition

**Figure 3:**
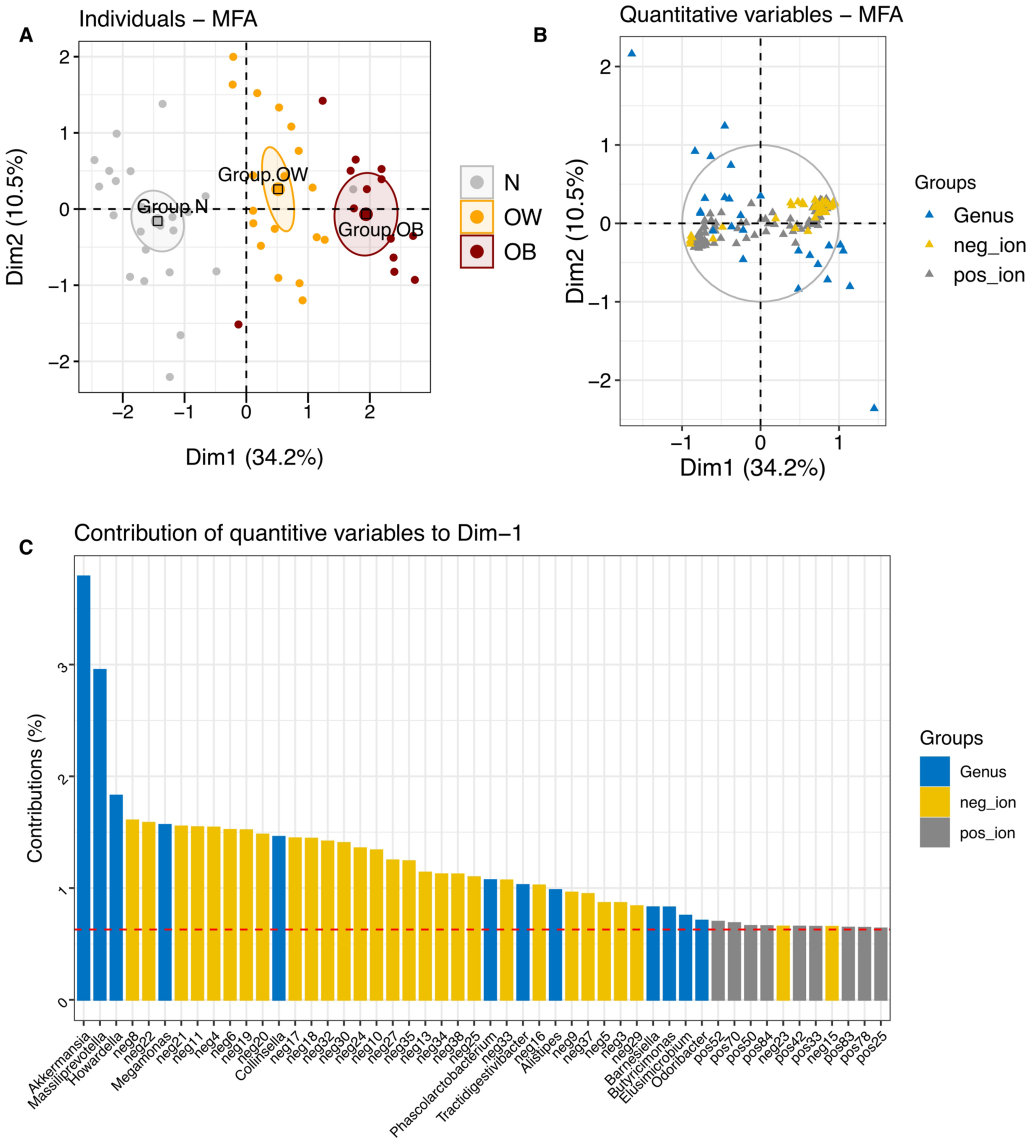
Multiple factor analysis (MFA) integrating gut microbiota and metabolite profiles across BMI groups. **(A)** Factor map showing individual profiles for each BMI group, with 95% confidence ellipses plotted on dimensions 1 and 2. **(B)** Correlation of quantitative variables [gut microbiota at the genus level (blue), negative-ion mode metabolites (yellow), and positive-ion mode metabolites (gray)] with MFA dimensions. **(C)** Contribution of quantitative variables to dimension 1. BMI groups: N = normal weight (*n* = 24); OW = overweight (*n* = 18); OB = obese (*n* = 13). pos_ion = positive-ion mode; neg_ion = negative-ion mode.

Multiple factor analysis (MFA) integrating gut microbiota abundance and fecal metabolite profiles revealed that inter-individual variations across BMI groups were primarily explained by metabolite profiles on Dimension 1 (34.2% explained variance), and by gut microbiota composition on Dimension 2 (10.5% explained variance) (Figure 3; Supplementary File S5). On Dimension 1, both positive-ion (cos^2^ = 0.76) and negative-ion (cos^2^ = 0.82) metabolites were major contributors to the observed variations. This was best illustrated by a distinct separation between the OB group (coordinate = 1.60, *p* < 0.0001) and the N group (coordinate = −1.77, *p* < 0.0001), reflecting contrasting metabolite profiles, especially in negative-ion mode (Supplementary Table S1). Several negative-ion compounds, including neg8, neg22, neg21, neg11, neg4, neg6, and neg19, were highly correlated with this dimension and enriched in the OB group. In contrast, neg17, neg18, neg30, and neg24 were associated with the N group. Although gut microbiota showed a high contribution (79.74%) to dimension 2, no single dimension (Dim 1 to Dim 4) adequately represented microbiota composition (summed cos^2^ < 0.5). Overall, the MFA demonstrated that BMI was more strongly associated with variations in fecal metabolite profiles than with gut microbiota composition in this study.

**Figure 4:**
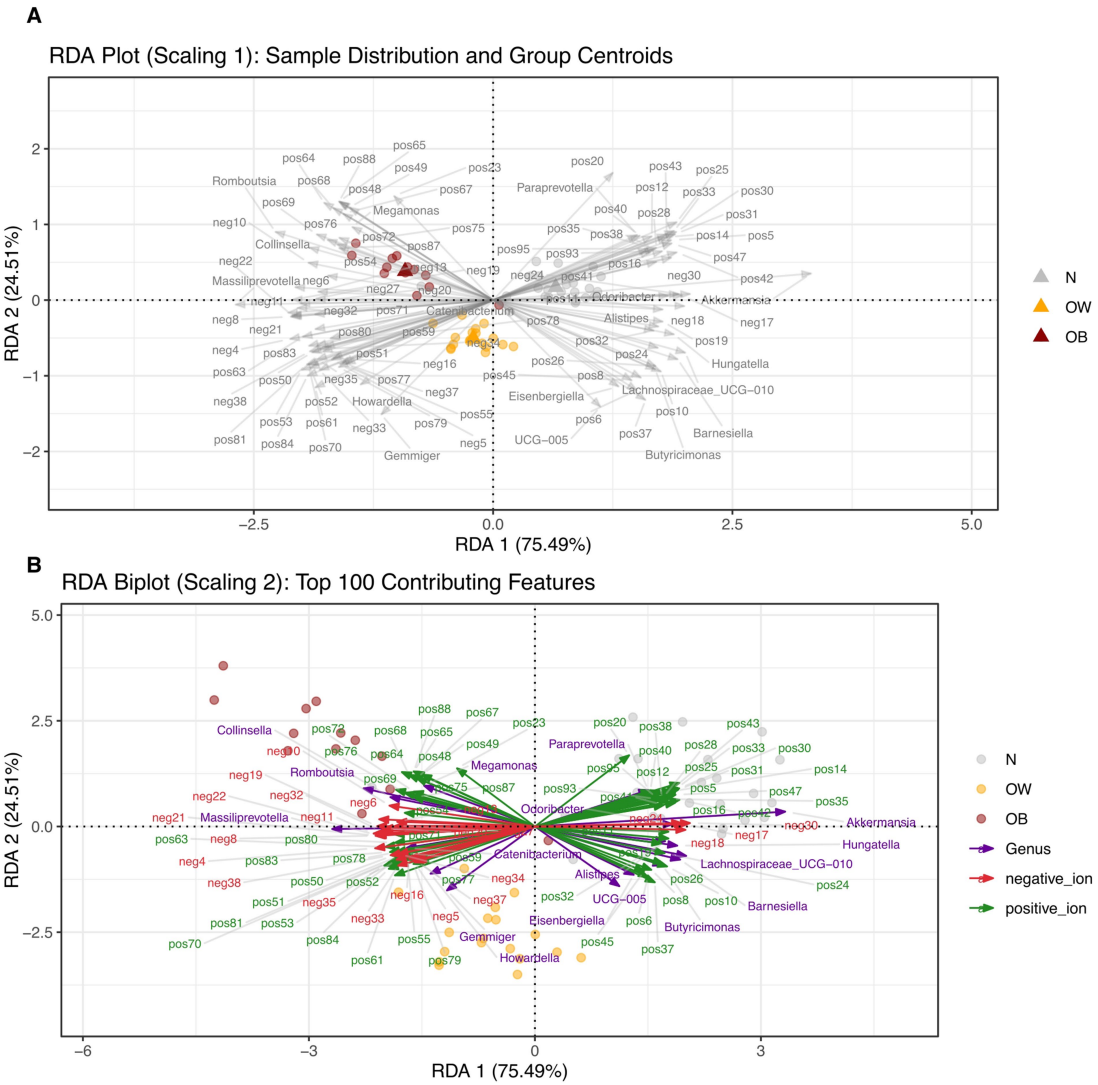
Redundancy analysis (RDA) showing the influence of BMI groups on gut microbiota and metabolite profile variation. **(A)** RDA biplots with type 1 scaling, where distances between samples represent similarity. Each triangle denotes the centroid of an explanatory variable (BMI group). **(B)** RDA biplots with type 2 scaling, where angles between arrows reflect correlations among variables. The analysis includes gut microbiota (144 genera) and metabolite profiles [positive-ion mode (*n* = 95) and negative-ion mode (*n* = 38)]. The significance of the constraints was assessed using an ANOVA-like permutation test (999 permutations). BMI groups: N = normal weight (*n* = 24); OW = overweight (*n* = 18); OB = obese (*n* = 13). positive_ion = positive-ion mode; negative_ion = negative-ion mode.

Furthermore, RDA revealed that BMI groups (X) significantly influenced metabolite profiles (Y), with the model yielding a high canonical correlation (*F_anova.cca_* = 30.33, *p* = 0.001) and a constrained proportion of 53.84% (variance in Y explained by X; adjusted *R*^2^ = 0.52). When gut microbiota composition at the genus level was included in the RDA model of metabolites, the model remained significant (*F_anova.cca_* = 4.92, *p* = 0.001), though the constrained proportion dropped substantially to 15.93% (adjusted *R*^2^ = 0.13). In the combined model, BMI explained 75.49% of the variation in RDA1 (*F_anova.cca_* = 7.44, *p* = 0.001) and 24.51% in RDA2 (*F_anova.cca_* = 2.41, *p* = 0.001). Distinct clustering of BMI groups was observed on scaling 1 (Figure 4A), where both metabolite features and gut microbiota showed an alignment with BMI group centroids, as indicated by arrow directionality. Among the top 100 contributing features, several positive-ion metabolites and bacterial genera, including *Collinsella*, *Romboutsia*, and *Meganomas*, shared vector directions in the OB group space and clustered near the group centroid. Conversely, some positive-ion metabolites and gut microbiota such as *Paraprevotella*, *Akkermansia*, and *Odoribacter* were clustered near the N group centroids. Many of the negative-ion metabolites also exhibited vector directions aligned with the OB and OW group spaces, while positive-ion features were enriched in the N group space. On scaling 2 (Figure 4B), both *Romboutsia* and *Collinsella*, associated with the OB group, shared directional similarity and showed strong correlations with several positive-ion metabolites including pos87, pos75, pos76, and pos69 (Supplementary Table S2). In contrast, Megamonas demonstrated strong correlations with pos68, pos67, pos64, pos88, pos65, pos48, pos49 in the OB group space. Within the N group space, *Paraprevotella* showed a strong correlation exclusively with positive-ion metabolites, whereas *Akkermansia*, positioned further from the N group centroid, correlated strongly with neg24 (Asn His Val), neg30 (3′?-Isopravastatin), *Odoribacter*, neg17 (1-[2-Fluoro-4-(methanesulfonyl)phenyl]-1,4-diazepane), and neg18 (Hoechst 33342). Collectively, these RDA results highlight a strong influence of BMI on fecal metabolite profiles, as well as associations between metabolite features and specific gut microbiota that varied by BMI group. Moreover, the relatively lower explanatory power of gut microbiota (genus level) in the integrated model indicated fewer interactions between these two dataset in this study.

We further examined the associations between metabolites (positive- and negative-ion modes) and gut microbiota at the genus level using HAllA. The analysis revealed no significant association patterns between these two datasets within each BMI group after *p*-value adjustment.

### 3.5 Shifts in association patterns between gut microbiota and predicted function (KOs) across BMI groups

**Figure 5:**
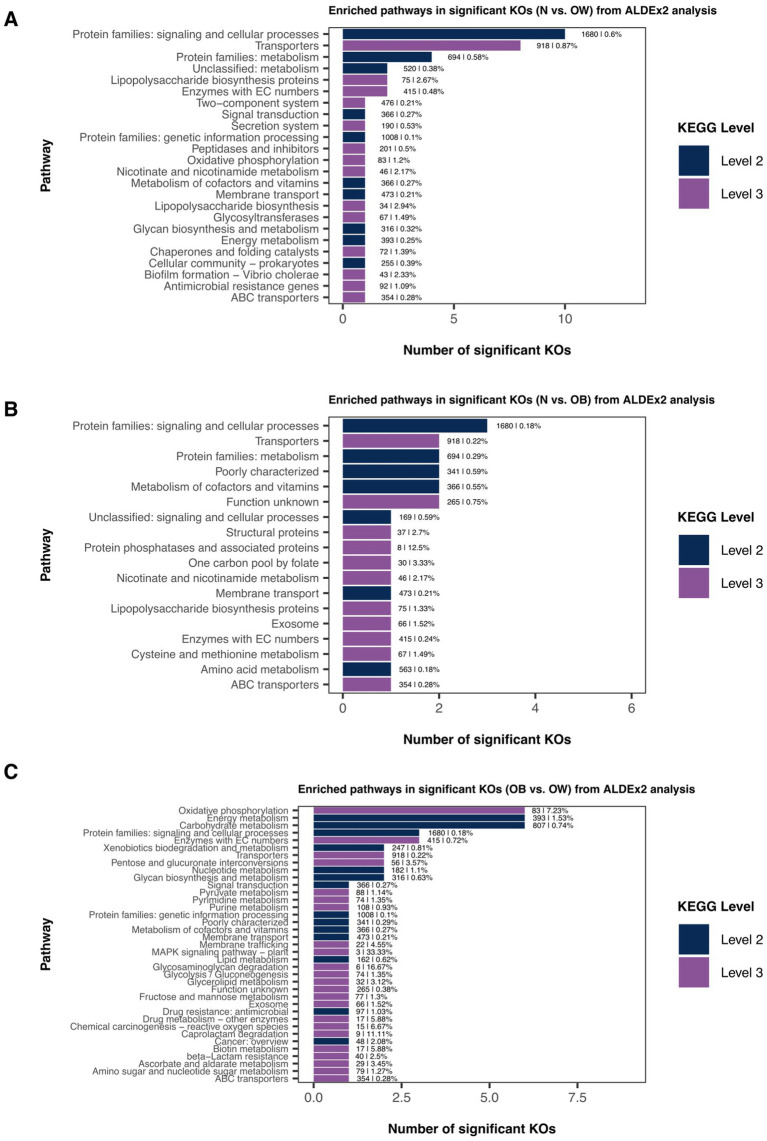
Pathway annotation of significantly differentially abundant KEGG Orthologs (KOs) between BMI groups based on ALDEx2 analysis. Bar plots showing enriched pathways among significantly different KOs identified by ALDEx2 for each group comparison: **(A)** N vs. OW, **(B)** N vs. OB, and **(C)** OB vs. OW. Pathway annotations are presented at KEGG level 2 (dark blue bars) and level 3 (purple bars). Bar labels indicate the total number of KOs and their proportional representation within each annotated pathway. BMI groups: N = normal weight (*n* = 30); OW = overweight (*n* = 20); OB = obese (*n* = 17).

Gut microbiota functional prediction identified a total of 5,819 KEGG genes across 54 KEGG level 2 pathways and 420 KEGG level 3 pathways (Supplementary File S6). Among these, K03088 (*rpoE*; RNA polymerase sigma-70 factor, ECF subfamily) was the most abundant KO across all BMI groups (Supplementary Figure S9). The top five most abundant level 2 pathways included *protein families: signaling and cellular processes* (16.77%), *protein families: genetic information processing* (10.06%), *carbohydrate metabolism* (8.06%), *protein families: metabolism* (6.93%), and *amino acid metabolism* (5.62%). At KEGG level 3, the most abundant pathways were *transporters* (9.16%), *two-component system* (4.75%), *enzymes with EC numbers* (4.14%), *ABC transporters* (3.53%), and *function unknown* (2.64%). Differential abundance analysis of KEGG orthologs (KOs) between BMI groups using ALDEx2 revealed 18, 9, and 17 differentially abundant KOs for N vs. OW, N vs. OB, and OB vs. OW, respectively (Supplementary Figures S10, S11). However, only differentially abundant KOs identified in N vs. OB comparisons remained statistically significant after *p*-value adjustment (*we.eBH* < 0.05). Mapping these differentially abundant KOs (*we.ep* < 0.05 or *we.eBH* < 0.05) to KEGG pathways revealed distinct enrichment patterns across ALDEx2 comparisons. In the N vs. OW comparison, *protein families: signaling and cellular processes* (level 2) and *transporters* (level 3) were the most enriched pathways (Figure 5A). In the N vs. OB comparison, *protein families: signaling and cellular processes* (level 2) was the most enriched (Figure 5B), while in the OB vs. OW comparison, the top three enriched pathways were *oxidative phosphorylation* (level 3), *energy metabolism* (level 2), and *carbohydrate metabolism* (level 2) (Figure 5C).

We further explored the associations between gut microbiota at the genus level and predicted functions (KOs) using HAllA. The analysis revealed dynamic shifts in association patterns across BMI groups (Supplementary Figure S12; Supplementary File S7). As BMI increased (N ⇢ OW ⇢ OB), the number of significant association clusters between gut microbiota and KOs declined markedly (*N* = 8,767; OW = 2,281; OB = 1,934). For example, the number of KOs significantly associated with major genera (*q* < 0.05, regardless of correlation strength), such as *Segatella* and *Bacteroides*, substantially decreased with increasing BMI (*N* = 1,709 and 927; OW = 523 and 523; OB = 314 and 120, respectively). *Segatella* also maintained strong associations with several abundant KOs ranked among the top 20 (Supplementary Figure S13). In contrast, OB-enriched genera including *Faecalibacterium* (OB = 191; *N* = 29), *Megamonas* (OB = 131; *N* = 8), *Collinsella* (OB = 74; *N* = 41), and *Brevundimonas* (OB = 59; *N* = 0) exhibited a notable increase in the number of associated KOs based on strong and significant HAllA clusters (*r* ≥ |0.7|, *q* < 0.05; Supplementary Figure S14). Conversely, *Phascolarctobacterium* showed relatively few KO associations in the OB group (OB = 5) compared to the N group (*N* = 245). Our results suggested a major reorganization in the functional interactions of the gut microbiome in obesity, characterized by fewer significant associations among dominant genera and increased coordination between OB-enriched genera and KOs. To visualize unique and shared associations across groups, we extracted significant and strong correlation clusters (excluding unclassified genera) from HAllA outputs (Spearman correlation coefficient *r* ≥ |0.7|, *q* < 0.05). This yielded 2,837 unique associations in the N group, 4,360 in OW and 4,234 in OB. Fewer associations were shared between the N and OB groups (1,160) compared to the N and OW groups (1,987), while 1,436 associations were shared between OW and OB. Among the top unique associations, *Segatella* was the dominant genus in the N group, showing strong negative correlations with most KOs. In contrast, *Ruthenibacterium* in the OW group demonstrated predominantly strong positive associations with KOs. In the OB group, genera such as *Blautia*, *Segatella*, *NK4A214 group*, and *Faecalibacterium* exhibited exclusively positive correlations with KOs. Additionally, OB-enriched genera like *Collinsella* and *Brevundimonas* also showed solely positive associations, whereas *Megamonas* displayed predominantly negative correlations with KOs. We also found that only one significantly different KO identified by ALDEx2 in the N vs. OB comparison overlapped with genus–KO associations detected by HAllA. This overlapping KO was K08281 (*pncA*; nicotinamidase/pyrazinamidase [EC:3.5.1.19, 3.5.1.-]), which was less abundant in the OB group. In contrast, greater overlap was observed in the N vs. OW (5 KOs) and OW vs. OB (4 KOs) comparisons. These findings suggest a progressive reduction in the connection between differentially abundant KOs and their associations with gut microbiota as BMI increases. In other words, changes in functional profiles may not directly correspond to specific microbiota–function relationships, particularly in higher BMI groups.

To further investigate the interactions between gut microbiota and predicted functions (KOs), we performed network analysis using the strong HAllA correlation clusters for each BMI group. Louvain clustering was applied to detect community structures and connection patterns between gut microbiota and KOs. The analysis revealed increased network modularity and decreased centralization in the OB group compared to the OW and N groups, indicating more distinct community structures than centralized interactions within the OB network (Supplementary File S7). These findings implied that in N and OW individuals, microbial functions were more diffusely interconnected, whereas in obese individuals, functional interactions became more compartmentalized and structured. *Segatella* and *Bosea* served as a dominant hub in the N and OW groups, respectively, each displaying the highest connectivity to KOs. However, in the obese (OB) group, the hub centrality of both *Segatella* and *Bosea* declined by more than half, and they were surpassed by *Megamonas*, which became the new dominant hub. This marked shift suggested a substantial reorganization of the gut microbiota–function interaction network in obesity, with central functional roles redistributed among different taxa.

### 3.6 Bridging gut microbiota and metabolite interactions through predicted microbial functions

Determining the associations between metabolites and predicted functions (KOs) using HAllA revealed dynamic shifts in association patterns across BMI groups (Supplementary Figure S15). The number of significant association clusters (*q* < 0.05) between metabolites and KOs declined with increasing BMI, with 467 clusters in the N group, 13 in the OW group, and 181 in the OB group. When focusing on strong associations (*r* ≥ |0.7|, *q* < 0.05), 22 out of 54 metabolites were enriched by more than 200 KO associations in the N group. Among these, neg34 and pos84 were the top two metabolites with the highest number of KO associations, each linked to 322 associations (Supplementary Table S3). In the OW group, pos30 and pos43 were the dominant KO-associated metabolites (Supplementary Figure S16A). Notably, pos30 also remained predominant in the OB group (Supplementary Figure S16B), associated with 169 KOs. In addition, all metabolite–KO associations in the OW group were exclusively positive. Our results suggested a decline in functional interactions between metabolites and KOs in overweight individuals compared to the normal group.

**Figure 6:**
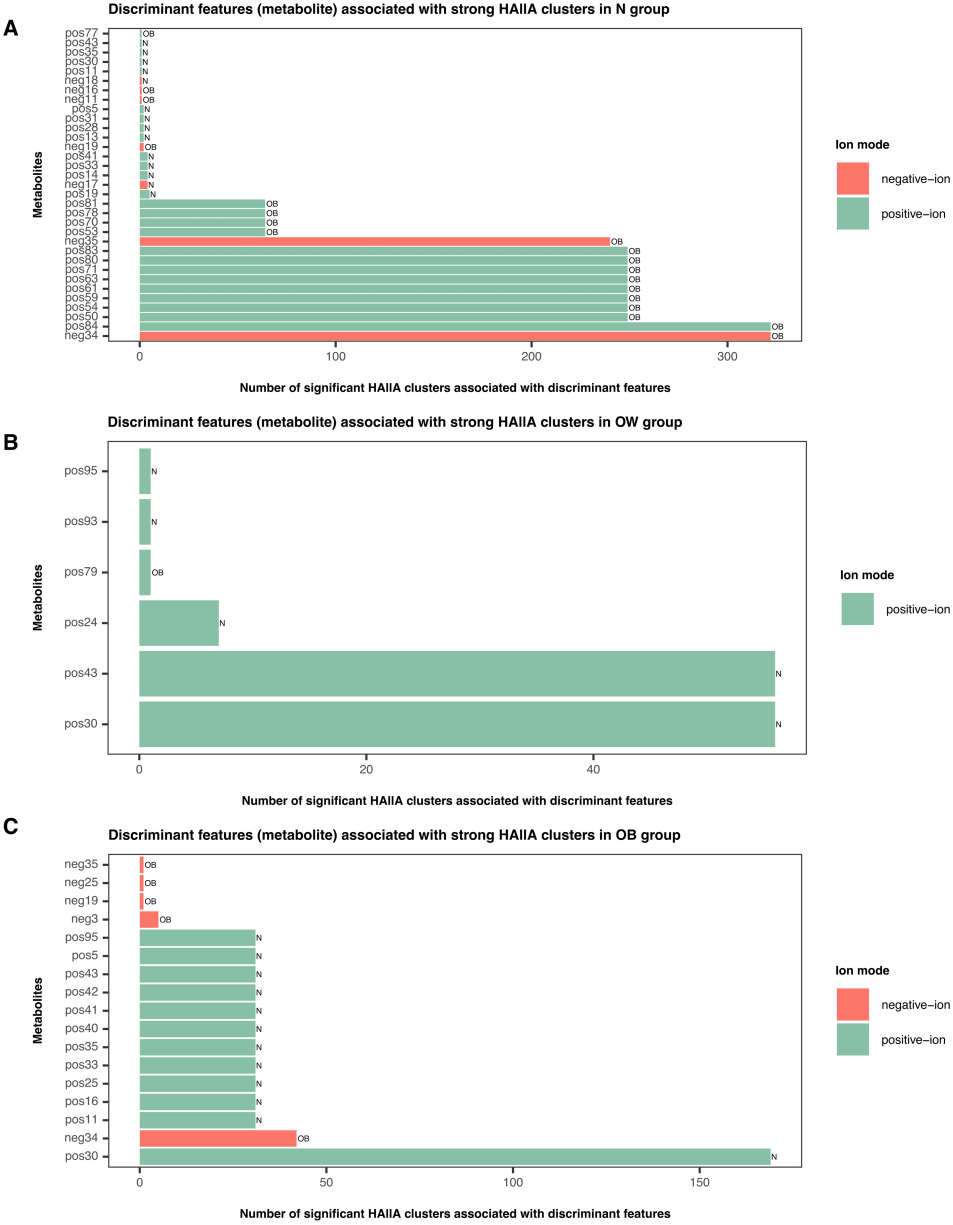
Discriminant metabolite features associated with strong HAllA clusters in each BMI group. Bar plots illustrate the number of significant HAllA clusters linked to discriminant metabolite features identified by the sPLS-DA tuned model (VIP > 1) in **(A)** N, **(B)** OW, and **(C)** OB groups. Green bars represent metabolites detected in positive-ion mode, while red bars represent those detected in negative-ion mode. Bar labels correspond to discriminant metabolites contributing to BMI group separation (VIP > 1) as determined by the sPLS-DA model. Full compound names are listed in Supplementary File S4. BMI groups: N = normal weight (*n* = 24); OW = overweight (*n* = 18); OB = obese (*n* = 13).

Furthermore, none of the differentially abundant KOs identified by ALDEx2 overlapped with the strong metabolite–KO associations detected by HAllA. By integrating significantly strong HAllA associations, differentially abundant metabolites between BMI groups, and discriminant features from the sPLS-DA tuned model (component 1; VIP > 1) revealed that OB-discriminant metabolites showed a high number of associations with KOs in the N group, whereas N-discriminant metabolites predominantly interacted with KOs in the OW and OB groups (Figure 6). This pattern suggested a contrasting enrichment profile of metabolite–KO associations across BMI groups, particularly between the N and OB groups.

Integrating metabolites and the stratified output from PICRUSt2 (KO abundance stratified by contributing ASVs at the genus level, KO|Genus) into HAllA revealed that the number of significant association clusters (*q* < 0.05) between metabolites and KO|Genus substantially declined in the N group compared to the higher BMI groups, with 6 clusters in the normal (N) group, 154 in the OW group, and 91 in the OB group (Supplementary File S9). In the OW group, pos17 (PG(22:6(4Z,7Z,11E,13Z,15E,19Z)-2OH(10S,17)/i-20:0)) was the major metabolite forming the highest number of clusters with KO|Genus (109 clusters), while pos23 (unclassified) and pos93 (Fumitremorgin B) shared 17 clusters. In the OB group, pos2 (Riboflavin, Vitamin B2) was the dominant cluster-associated metabolite (57 clusters), while pos30 formed 6 clusters. Both pos17 and pos2 served as major hubs in the interaction networks of the OW and OB groups, respectively. More than 85% of the significant associations were strong across BMI groups. Further analysis of replicated associations based on shared KO|Genus patterns (i.e., KO|Genus groups that appeared with the same set of metabolites) revealed that only K00179|*Collinsella*, an OB-enriched genus, formed a strong positive association with pos30 (*r* = 1, *q* < 0.0001), and this association was unique to the OB group. None of the other OB-enriched genera, including *Faecalibacterium*, *Brevundimonas*, *Megamonas*, and *Phascolarctobacterium*, were KO-contributing taxa linked to any metabolites. Most genera (KO|Genus) associated with metabolites were not among the significantly abundant taxa across BMI groups. In addition, none of the strong associations were shared between the N and OW groups or between the N and OB groups. Twenty-eight associations were shared between the OW and OB groups, all involving pos30, pos43, and pos95 (Ala Phe Pro Glu). Specifically, pos30 and pos95 were associated with KO-contributing *Bacteroides*, while pos43 interacted with both KO-contributing *Bacteroides* and UCG-002. None of the differentially abundant KOs identified by ALDEx2 overlapped with the strong metabolite–KO|Genus associations detected by HAllA. Integration of significantly strong HAllA associations, differentially abundant metabolites between BMI groups, and discriminant features from the sPLS-DA tuned model (component 1; VIP > 1) yielded a similar pattern to the HAllA metabolite-KO associations. OB-discriminant metabolites were predominantly associated with KO|Genus in the N group, while N-discriminant metabolites showed more frequent associations with KO|Genus in the OW and OB groups (Supplementary Figure S17).

Collectively, our study demonstrated that gut microbiota interacted with metabolites primarily through functional bridging mechanisms rather than through direct associations. Interaction profiles involving metabolite variables were more similar between the higher BMI groups (OW and OB), highlighting a shared pattern of microbiome-metabolite crosstalk in individuals with elevated BMI. Significantly abundant features (gut microbiota or KOs) contributed less to the metabolite–KO or metabolite–KO|Genus interaction space across BMI groups. Pos30 served as a key metabolite in both OW and OB groups. Its strong association with the OB-enriched genus, *Collinsella*, via K00179 (iorA; indolepyruvate ferredoxin oxidoreductase, alpha subunit [EC:1.2.7.8]) appeared to represent an OB-specific functional signature in this study.

## 4 Discussion

This study aimed to investigate gut microbiota and metabolite profiles of obesity in Thai school-age children, hypothesizing changes in gut microbiota composition and metabolite abundance as BMI increased. As speculated, we observed significant differences in gut microbiota abundance across BMI groups. BMI had an even greater impact on metabolite profiles. Our study also highlighted distinct alterations in microbiota–function associations linked to obesity.

Previous studies in Chinese school-aged children have reported significantly lower alpha diversity (ACE and Chao1 indices) in obese individuals compared to normal-weight children, despite inconsistent findings regarding beta diversity based on unweighted and weighted UniFrac distance metrics (Jiang et al., 2022; Li et al., 2024). Similarly, broader evidence has suggested that obesity is generally associated with reduced gut microbial diversity (Enache et al., 2024). In contrast, our study did not identify significant differences in any alpha diversity indices or in abundance-based beta diversity metrics, such as the weighted UniFrac and Bray–Curtis distance matrices. However, significant differences were observed in the unweighted UniFrac distance matrix between the N and OB groups. These results implied that differences in microbial community between N and OB groups may be driven by variations in microbial presence or absence, particularly across individuals at opposite ends of the BMI status. Although our results differed from some previous studies with respect to diversity metrics, they supported the gut microbiota community shifts associated with obesity, particularly at lower taxonomic levels.

The gut microbiota composition in OB individuals was more distinct from that of N than from the OW group, with differences primarily driven by changes in the relative abundance of specific taxa, particularly at the genus level. *Faecalibacterium*, *Megamonas*, *Collinsella*, *Brevundimonas*, *Phascolarctobacterium* were among the most prevalent genera in the OB group. The genus *Collinsella* has consistently shown higher abundance in individuals with obesity across studies ranging from young children (*n* = 63) to adults (*n* = 128) (Jiang et al., 2022; Companys et al., 2021). *Megamonas* and *Phascolarctobacterium* were also enriched in obese school-aged children (*n* = 51) (Chen et al., 2020); however, in that same study, *Collinsella* was more abundant in normal-weight children. While these findings highlight distinct microbial patterns associated with obesity, inter-study variations warrant further investigation in larger cohorts. This may help clarify whether the observed overlaps in obesity-associated taxa between children and adults could support biomarker discovery for obesity, regardless of age. Furthermore, several beneficial taxa such as *Ruthenibacterium*, *Lachnospiraceae* UCG-010, *Paludicola*, and *Eisenbergiella* were least abundant in the OB group. These bacteria are known as SCFA producers (Hitch et al., 2025; Hartinger et al., 2022; Akasaka et al., 2003; Vinelli et al., 2022). The abundances of the latter two genera were also depleted in animal models undergoing high-fat and high-sugar diet treatments for 18 weeks (Pessoa et al., 2023).

Previous evidence has shown that obesity is associated with elevated oxidative stress levels (Masschelin et al., 2020). In this study, six significantly enriched KOs corresponding to different subunits of the V/A-type H^+^/Na^+^-transporting ATPase (K02117, K02118, K02123, K02120, K02124, K02121), within the oxidative phosphorylation pathway (level 3), were identified in the OB group. All six KOs were consistently associated with the same 27 metabolites (Supplementary File S6). However, none of these KOs were linked to *Faecalibacterium*, despite its enrichment in OB subjects. This finding is consistent with our previous observation (Gruneck et al., 2020), where we highlighted that *F. prausnitzii*, a representative butyrate-producing species, may serve as a microbial indicator of host physiological status, particularly in the context of obesity. Instead, KO-contributing taxa identified by stratified PICRUSt2 analysis included other SCFA-producing genera such as *Butyricimonas* and *Lachnospiraceae* UCG-010, both of which were significantly reduced in OB compared to N. Interestingly, certain SCFA-producing genera including *Prevotellaceae Ga6A group* and *Ruminococcus* did not show significant differences in abundance between OB and N groups, implying that these genera may retain compositional stability under increased BMI conditions and support community resilience. Although *Faecalibacterium* was not directly associated with the KO–metabolite interactions, the sustained abundance of *Faecalibacterium* in obese individuals may be supported indirectly through microbial community interactions, particularly with more stable SCFA producers (Maioli et al., 2021). Collective evidence also suggests *Faecalibacterium* species vary in oxidative stress responses (Martín et al., 2023), implying that its abundance in the OB group may reflect strain-level adaptations and ecological interactions within the gut. The potential role of these bacteria in obesity warrants validation at higher taxonomic resolution. In this study, gut microbiota were profiled only from the phylum to genus levels, limiting differentiation of functionally distinct species or strains and preventing links between taxa and functional genomic information (Johnson et al., 2019). Future studies integrating shotgun metagenomics with species-specific qPCR could enhance taxonomic resolution, detect divergence at finer scales (e.g., *Faecalibacterium* vs. *F. prausnitzii*), and confirm key taxa and their associations with functional and metabolite profiles relevant to obesity. Furthermore, the consistent association of the six oxidative phosphorylation-related KOs with the same 27 metabolites in HAllA analysis points toward specific metabolite–KO interactions, emphasizing the need for targeted metabolomic investigations, particularly focused on SCFAs, to further elucidate microbe–metabolite networks and their roles in gut community dynamics under obese conditions. However, the predicted KOs from PICRUSt2 in this study were putative, derived from partial 16S amplicon-based functional predictions, and could not resolve microbial function at the strain level (Douglas et al., 2020). While these predictions provide an overview of potential functions, they do not reflect actual gene expression or account for horizontal gene transfer. Future studies incorporating metatranscriptomic or metaproteomic analyses could validate these functional shifts, integrate them into a comprehensive multi-omics framework, and clarify how gut microbiota interact within their communities to influence host health.

Previous studies profiling fecal metabolites have reported elevated levels of certain amino acids, SCFAs, and/or other metabolites commonly associated with overweight and/or obesity in both children (Jaimes et al., 2021) and adults (Cui et al., 2021). These trends are consistent with our findings, which show that approximately 80% of distinct negative-ion features were more enriched in both the OW and OB groups. These two groups also appeared metabolically closer to each other than to the N group. Several enriched metabolites were short amino acid residues (e.g., neg 4: Ala-Lys-Pro-Gln; neg 7: Lys-Asp-Ser; neg 14: Ala-Asp-Pro-Asp; neg 22: Phe-Glu-Arg). The increased abundance of these metabolites in higher BMI groups suggests that the negative-ion metabolite profile identified in this study may serve as a promising target for further investigation into biomarkers relevant to BMI-related metabolome characterization.

Accumulated evidence has demonstrated that imbalances in gut microbiota composition and function can promote the progression of host obesity by disrupting metabolic processes, including energy uptake and expenditure, as well as lipid synthesis and storage (Cheng et al., 2022; Yarahmadi et al., 2024). In our study, linking gut microbiota to their encoded functions revealed a progressive loss of microbiota–function connectivity with increasing BMI. Dominant genera such as *Segatella* and *Bacteroides* lost many of their functional associations in the OB groups, while OB-enriched genera (e.g., *Faecalibacterium*, *Megamonas*, *Collinsella*, *Brevundimonas*) gained more associations with KOs. This decline in functional connectivity may reflect a progressive disruption of microbial functional integration in higher BMI states, potentially accompanied by a compensatory shift in functional roles among different taxa. Further research is warranted to clarify the mechanisms underlying these microbiota–function shifts, ideally through whole-genome sequencing of gut microbes combined with gene expression profiling and the quantification of relevant parameters such as lipid levels and targeted metabolite profiles.

Linking gut microbiota to metabolite profiles showed no direct relationship between these two variables in our study. This absence of association aligns with findings from a previous study involving school-aged children with varying BMI status, which similarly reported no significant link between gut microbiota composition and metabolite profiles (Jaimes et al., 2021). The lack of direct associations in our study may reflect (1) reliance on *in silico* functional mediation, (2) a relatively small sample size, and (3) the presence of fecal metabolites originating from non–gut microbiota sources. Future studies integrating targeted metabolomic approaches with alternative analytic frameworks, such as sparse Canonical Correlation Analysis, may help identify key features driving cross-domain associations. Furthermore, none of the differentially abundant KOs formed strong associations with metabolites, and most KO|Genus features linked to metabolites were not among the significantly abundant taxa across BMI groups. Instead, our analysis revealed that microbiome–metabolite interactions were predominantly mediated through microbial functions (KOs), rather than direct taxonomic associations. These findings highlighted the importance of incorporating predicted functional profiles when investigating microbiome-metabolite crosstalk, particularly with respect to BMI-related gut microbial profiles. This function-centered approach provides a deeper understanding of how BMI influences the triangular relationship among gut microbiota, their functions, and metabolites, independent of taxonomic abundance at the genus level.

A key strength of our study lies in the comprehensive profiling of gut microbiota and non-targeted metabolites, as well as their functional interactions, among Thai school-aged children. However, several limitations should be acknowledged. As this was a cross-sectional study with a relatively small sample size, particularly for metabolomics subset analyses (*n* = 55) such as HAllA clustering, the statistical power may have been insufficient to detect true biological associations, thereby limiting the strength of our conclusions. Larger cohorts are needed to robustly validate key findings, including metabolite biomarkers and microbiota–function–metabolite relationships, in independent samples. The lack of dietary, physical activity, and socioeconomic data also constrained interpretation, as these factors likely influenced gut microbiota and metabolite profiles in the children but could not be accounted for in this study. Future research should incorporate self-reported dietary records and adjust analyses for energy intake and fiber consumption, which would strengthen the validation of their contributions to the gut microbiota and metabolome in children. In addition, the non-targeted metabolomic approach used in our study left many discriminant metabolites unclassified. Integrating non-targeted and targeted analyses (e.g., short-chain and branched-chain fatty acids), MS/MS fragmentation, and whole-genome sequencing could improve metabolite identification, clarify key taxa, and provide deeper insights into microbe-metabolite interactions and their functional roles in shaping gut microbial communities in obesity.

## 5 Conclusion

This study revealed significant differences in gut microbiota and non-targeted metabolite profiles across BMI groups in school-aged children. Several genera, including *Faecalibacterium*, *Collinsella*, *Megamonas*, *Brevundimonas*, and *Phascolarctobacterium*, were enriched in the OB group. BMI exerted a stronger influence on metabolite profiles, with negative-ion mode metabolites might be valuable targets for future research aimed at obesity characterization. Shifts in microbiota–function associations, particularly the loss of functional connections among dominant genera and compensatory gains among OB-enriched genera, warrant further investigation into their mechanistic roles under host metabolic alterations. Importantly, associations between gut microbiota, metabolites, and predicted functions were primarily explained via KO-contributing taxa, suggesting that microbiome–metabolite interactions may be mediated through functional pathways rather than direct taxon–metabolite correlations. This highlights the importance of a functional triangle framework in understanding host–microbe–metabolite crosstalk in obesity. Overall, these findings pave the way for targeted strategies to support early intervention and prevention of pediatric obesity in Thailand, enabling more effective, population-specific management approaches.

## Data Availability

The 16S rRNA gene sequences of the fecal gut microbiome generated in this study have been deposited in the NCBI Sequence Read Archive (SRA) (https://www.ncbi.nlm.nih.gov/sra) under BioProject accession number PRJNA1276713 (BioSample accession numbers SAMN49082378–SAMN49082444).
