# Supplementary material for: Gut microbiome and fecal metabolite profiles in obese school-aged children from Northern Thailand
Source: Front Microbiol. 2025 Sep 11;16:1657839. doi: 10.3389/fmicb.2025.1657839 (PMC12461226; doi:10.3389/fmicb.2025.1657839)
Supplement: Supplementary file 1 [file Data_Sheet_1.PDF]

Gut Microbiome and Fecal Metabolite Profiles in Obese School-Aged Children from Northern Thailand

Phatthanaphong Therdtatha[a], Lucsame Gruneck[b], Poramet Nachalam[b, c], Vasana Jinatham[b, d], Kritsakorn Saninjuk[b, d], Jiro Nakayama[e], Siam Popluechai[b, d*]

[a]Specialized Research in Microbiome and Metabolome for Health Laboratory, Division of Biotechnology, Faculty of Agro-Industry, Chiang Mai University, Chiang Mai, Thailand

[b]Gut Microbiome Research Group, Mae Fah Luang University, Muang, Chiang Rai, Thailand

[c]Scientific and Technological Instruments Center, Mae Fah Luang University, Chiang Rai, Thailand

[d]School of Science, Mae Fah Luang University, Muang, Chiang Rai, Thailand

[e]Laboratory of Microbial Technology, Division of Applied Molecular Microbiology and Biomass Chemistry, Department of Bioscience and Biotechnology, Faculty of Agriculture, Graduate School, Kyushu University, Fukuoka, Japan

***Correspondence:** Siam Popluechai, siam@mfu.ac.th

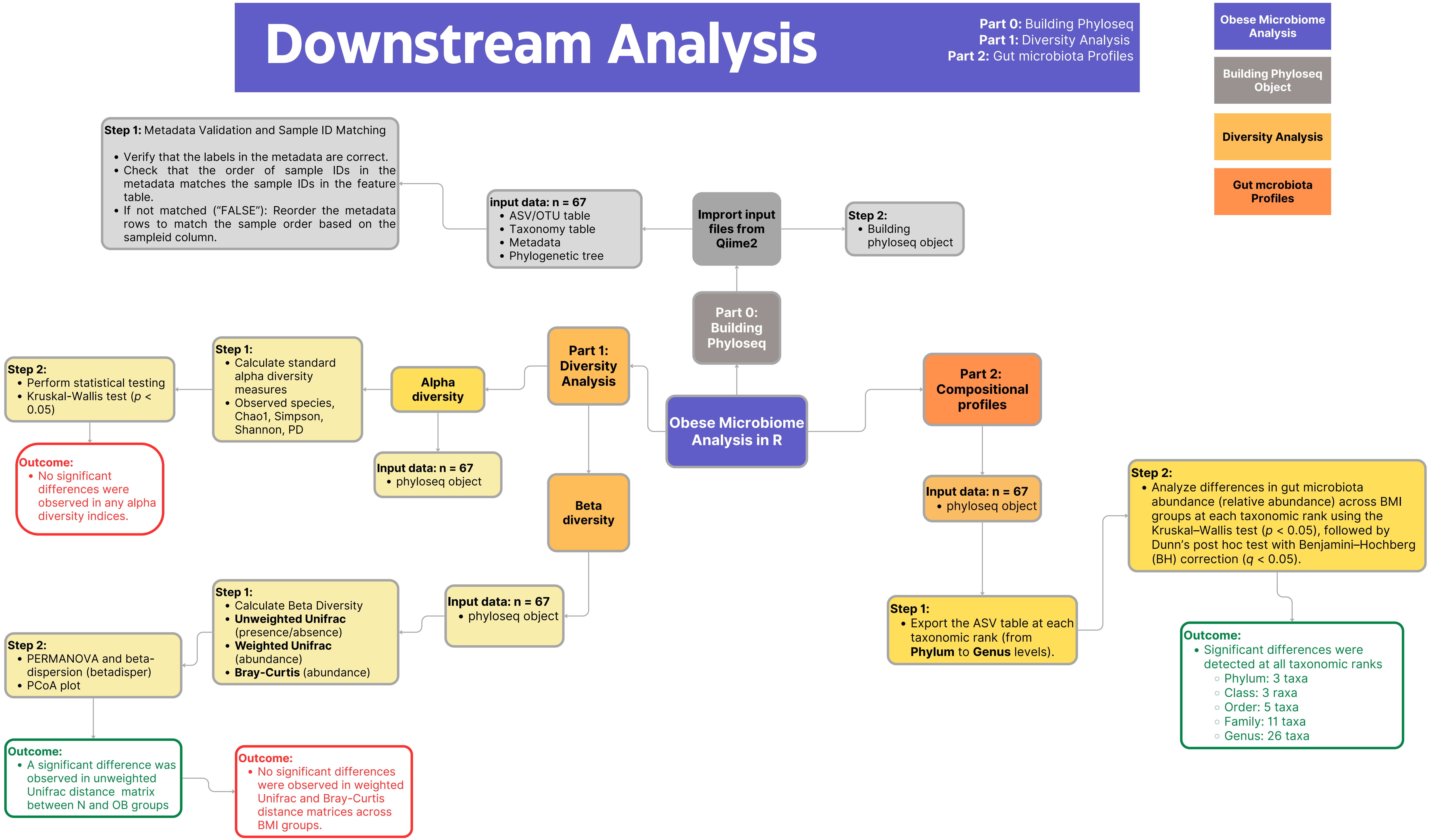
Downstream Analysis
Part 0: Building Phyloseq
Part 1: Diversity Analysis
Part 2: Gut microbiota Profiles
Obese Microbiome Analysis
Building Phyloseq Object
Diversity Analysis
Gut mcrobiota Profiles
Step 1: Metadata Validation and Sample ID Matching
• Verify that the labels in the metadata are correct.
• Check that the order of sample IDs in the metadata matches the sample IDs in the feature table.
• If not matched ("FALSE"): Reorder the metadata rows to match the sample order based on the sampleid column.
input data: n = 67
• ASV/OTU table
• Taxonomy table
• Metadata
• Phylogenetic tree
Imprort input files from Qiime2
Step 2:
• Building phyloseq object
Part 0: Building Phyloseq
Obese Microbiome Analysis in R
Part 1: Diversity Analysis
Part 2: Compositional profiles
Alpha diversity
Step 1:
• Calculate standard alpha diversity measures
• Observed species, Chao1, Simpson, Shannon, PD
Step 2:
• Perform statistical testing
• Kruskal-Wallis test (p < 0.05)
Outcome:
• No significant differences were observed in any alpha diversity indices.
Input data: n = 67
• phyloseq object
Beta diversity
Input data: n = 67
• phyloseq object
Step 1:
• Calculate Beta Diversity
• Unweighted Unifrac (presence/absence)
• Weighted Unifrac (abundance)
• Bray-Curtis (abundance)
Step 2:
• PERMANOVA and beta-dispersion (betadisper)
• PCoA plot
Outcome:
• A significant difference was observed in unweighted Unifrac distance matrix between N and OB groups
Outcome:
• No significant differences were observed in weighted Unifrac and Bray-Curtis distance matrices across BMI groups.
Input data: n = 67
• phyloseq object
Step 1:
• Export the ASV table at each taxonomic rank (from Phylum to Genus levels).
Step 2:
• Analyze differences in gut microbiota abundance (relative abundance) across BMI groups at each taxonomic rank using the Kruskal–Wallis test (p < 0.05), followed by Dunn's post hoc test with Benjamini–Hochberg (BH) correction (q < 0.05).
Outcome:
• Significant differences were detected at all taxonomic ranks
◦ Phylum: 3 taxa
◦ Class: 3 raxa
◦ Order: 5 taxa
◦ Family: 11 taxa
◦ Genus: 26 taxa

Downstream Analysis

Part 3: Metabolite Profiles

Obese Microbiome Analysis

Metabolite Analysis

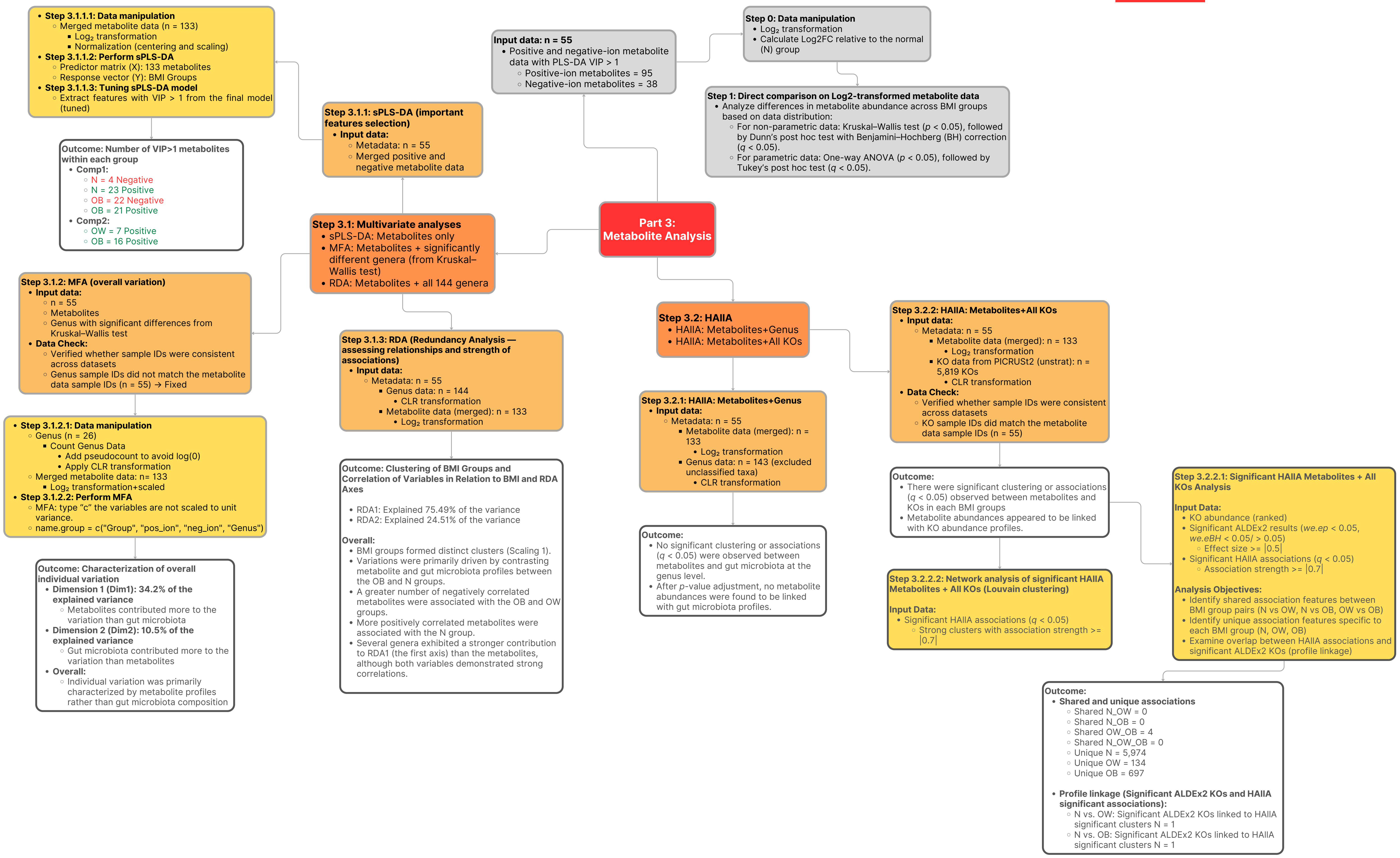

Downstream Analysis

Part 4: Functional Profiles

Obese Microbiome Analysis

Functional Profiles

Part 4: Functional Profiles

Input data: n = 67
- PICRUSt2 output (unstrat)
  - pred_metagenome_unstrat.tsv

Step 1: KEGG database mapping
- **Input:** KO ID (5,819 KOs)
- **Process:** Perform mapping using a Python script.

Step 4.1: ALDEx2 analysis
- **Input data:**
  - KO abundance data+add "pseudocount" (+1) for aldex.clr
  - Metadata (n =67)

Outcome:
- N vs OW:
  - Filtering conditions: *we.ep* < 0.05 & effect >= |0.5|
  - 18 significant KOs
- N vs OB:
  - *we.eBH* < 0.05 & effect >= |0.5|
  - 9 significant KOs
- OB vs OW:
  - *we.ep* < 0.05 & effect >= |0.8|
  - 17 significant KOs
- Note:
  - we.ep (Welch's t-test *p*-value) and we.eBH (Welch's t-test *q*-value)
  - Significant KOs were not among the top 100 KOs

Step 4.2: HAllA
- HAllA: Genus+All KOs

Step 4.2.1: HAllA: Genus+All KOs
- **Input data:**
  - Metadata: n = 67
    - Genus data: n = 143 (excluded unclassified taxa)
      - CLR transformation
    - KO data from PICRUSt2 (unstrat): n = 5,819 KOs
      - CLR transformation
- **Data Check:**
  - Verified whether sample IDs were consistent across datasets
  - Genus sample IDs did match the KO data sample IDs

Outcome:
- There were significant clustering or associations (*q* < 0.05) observed between genus and KOs in each BMI groups
- Gut microbiota abundances at the genus level appeared to be linked with KO abundance profiles.

Step 4.2.2: Network analysis of significant HAllA Genus + All KOs (Louvain clustering)

Input Data:
- Significant HAllA associations (*q* < 0.05)
  - Strong clusters with association strength >= |0.7|

Step 4.2.3: Significant HAllA Genus + All KOs Analysis

Input Data:
- KO abundance (ranked)
- Significant ALDEx2 results (*we.ep* < 0.05, *we.eBH* < 0.05/ > 0.05)
  - Effect size >= |0.5|
- Significant HAllA associations (*q* < 0.05)
  - Association strength >= |0.7|

Analysis Objectives:
- Identify shared association features between BMI group pairs (N vs OW, N vs OB, OW vs OB)
- Identify unique association features specific to each BMI group (N, OW, OB)
- Examine overlap between HAllA associations and significant ALDEx2 KOs (profile linkage)

Outcome:
- **Shared and unique associations**
  - Shared N_OW = 3,559
  - Shared N_OB = 2,042
  - Shared OW_OB = 2,603
  - Shared N_OW_OB = 4,257
  - Unique N = 2,837
  - Unique OW = 4,360
  - Unique OB = 4,234
- **Profile linkage (Significant ALDEx2 KOs and HAllA significant associations):**
  - There were significant ALDEx2 KOs linked to HAllA significant clusters across BMI group pairs (N vs OW, N vs OB, OW vs OB)

Step 4.3: Enriched Pathway level 2 and 3 analysis
- Metadata: n = 67
  - Mapped KO KEGG output
  - Significant ALDEx2 KOs

Step 4.3.1: Summarize pathway Level 2 and Level 3
- **Process:**
  - Count the total number of KOs in each Level 2 and Level 3 pathway
  - Calculate the proportion of KOs within each pathway

- **Step 4.3.2: Enriched pathways in significant KOs from ALDEx2 analysis**
  - Count significant KOs and total KOs per pathway Level 2
  - Count significant KOs and total KOs per pathway Level 3
  - Calculate the proportion of significant KOs within each pathway at Level 2 and Level 3

Downstream Analysis

Part 5: Integration Profiles

Obese Microbiome Analysis

Integration Profiles

Input data: n = 67
- PICRUSt2 output (strat)
  - pred_metagenome_strat.tsv
    - KO|Microbe

Step 0: Data manipulation
- **Process:**
  - Assigned taxonomy on Strat data
  - Filtered only significant ALDEx2 KOs from the stratified data (1,048575 observations) for integration with metabolite data in each BMI group.
  - Remaining:
    - N = 1,585 (Strat)
    - OW = 6,015 (Strat)
    - OB = 5,284 (Start)

Part 5: Integration Profiles (Metabolites+PICRUSt2 Strat (KO|Genus)

Step 5.1: HAllA
- HAllA: Metabolites+Strat (KO|Genus)

Step 5.1.1: HAllA:Metabolites+Strat (KO|Genus)
- **Input data:**
  - Metadata: n = 55
    - Strat data: filtered only significant ALDEx2 KOs
      - CLR transformation
    - Merged metabolite data: n= 133
      - Log_2 transformation+scaled
- **Data Check:**
  - Verified whether sample IDs were consistent across datasets
  - Strat sample IDs did match the metabolite data sample IDs

Outcome:
- There were significant clustering or associations ($q < 0.05$) observed between Strat (KO|Genus) and metabolites in each BMI groups
- KO|Genus abundances appeared to be linked with metabolites.

Step 5.1.2: Network analysis of significant HAllA Strat + Metabolites (Louvain clustering)

Input Data:
- Significant HAllA associations ($q < 0.05$)
  - Strong clusters with association strength >= |0.7|

Step 5.1.3: Significant HAllA Metabolites+Strat (KO|Genus)Analysis

Input Data:
- KO abundance (ranked)
- Significant ALDEx2 results (*we.ep* < 0.05, *we.eBH* < 0.05/ > 0.05)
  - Effect size >= |0.5|
- Significant HAllA associations ($q < 0.05$)
  - Association strength >= |0.7|

Analysis Objectives:
- Identify shared association features between BMI group pairs (N vs OW, N vs OB, OW vs OB)
- Identify unique association features specific to each BMI group (N, OW, OB)
- Examine overlap between HAllA associations and significant ALDEx2 KOs (profile linkage)
- Determine replicate KO patterns based on shared X_features (metabolites) as to group KOs that appear with the same set of X_features (metabolites)

Outcome:
- **Shared and unique associations**
  - Shared N_OW = 0
  - Shared N_OB = 0
  - Shared OW_OB = 28
  - Shared N_OW_OB = 0
  - Unique N = 120
  - Unique OW = 491
  - Unique OB = 1,135
- **Profile linkage (Significant ALDEx2 KOs and HAllA significant associations):**
  - No significant ALDEx2 KOs linked to HAllA significant clusters across BMI group pairs (N vs OW, N vs OB, OW vs OB)
