# Supplementary material for: Gut microbiome and fecal metabolite profiles in obese school-aged children from Northern Thailand
Source: Front Microbiol. 2025 Sep 11;16:1657839. doi: 10.3389/fmicb.2025.1657839 (PMC12461226; doi:10.3389/fmicb.2025.1657839)
Supplement: Supplementary file 2 [file Data_Sheet_2.PDF]

Gut Microbiome and Fecal Metabolite Profiles in Obese School-Aged Children from Northern Thailand

Phatthanaphong Therdtatha[a], Lucsame Gruneck[b], Poramet Nachalam[b, c], Vasana Jinatham[b, d], Kritsakorn Saninjuk[b, d], Jiro Nakayama[e], Siam Popluechai[b, d*]

[a]Specialized Research in Microbiome and Metabolome for Health Laboratory, Division of Biotechnology, Faculty of Agro-Industry, Chiang Mai University, Chiang Mai, Thailand
[b]Gut Microbiome Research Group, Mae Fah Luang University, Muang, Chiang Rai, Thailand
[c]Scientific and Technological Instruments Center, Mae Fah Luang University, Chiang Rai, Thailand
[d]School of Science, Mae Fah Luang University, Muang, Chiang Rai, Thailand
[e]Laboratory of Microbial Technology, Division of Applied Molecular Microbiology and Biomass Chemistry, Department of Bioscience and Biotechnology, Faculty of Agriculture, Graduate School, Kyushu University, Fukuoka, Japan

* **Correspondence:** Siam Popluechai, siam@mfu.ac.th

Metabolomic Analysis of Fecal Samples Using UHPLC-QTOF/MS

Instrument: UHPLC 1290 infinity ii / 6545B QTOF/MS system

Column: Agilent Poroshell 120 EC-C18 2.1*150mm,2.7 um

Formic acid (*Thermo* Scientific Pierce *Formic Acid For LCMS)*

ACN (Acetonitrile, BAKER ANALYZED® for LC-MS)

Water (Water, BAKER ANALYZED® for LC-MS)

UHPLC conditions:

Injection volume: 1 µl

Column Temperature: 35 °C

Gradient program:

Mobile Phase A: 0.1% Formic acid in water

Mobile Phase B: 0.1 % Formic acid in Acetonitrile

Flow rate: 0.2 ml/min.

**Time (minute)**	**% B**
0	5
1	5
10	17
13	17
20	95
25	95
27	5
35	5

UHPLC QTOF/MS instrument parameters

Ion source temperature	
Parameter	Set point
Gas temperature	300 °C
Gas Flow	10 L/min.
Nebulizer	35 psig
Sheath gas temp.	350 °C
Sheath Gas Flow	11 L/min.
Ion optic	
Vcap	3500 V
Nozzle voltage	1000 V
Fragmentation	175 V
Skimmer 1	65 V
Octopole RF	750 V
Acquisition mode	
Min range (m/z)	100
Max range (m/z)	1100
Acquisition rate (spectrum/min.)	2
