# Supplementary material for: Gut microbiome and fecal metabolite profiles in obese school-aged children from Northern Thailand
Source: Front Microbiol. 2025 Sep 11;16:1657839. doi: 10.3389/fmicb.2025.1657839 (PMC12461226; doi:10.3389/fmicb.2025.1657839)
Supplement: Supplementary file 3 [file Data_Sheet_3.PDF]

Gut Microbiome and Fecal Metabolite Profiles in Obese School-Aged Children from Northern Thailand

Phatthanaphong Therdtatha[a], Lucsame Gruneck[b], Poramet Nachalam[b, c], Vasana Jinatham[b, d], Kritsakorn Saninjuk[b, d], Jiro Nakayama[e], Siam Popluechai[b, d*]

[a]Specialized Research in Microbiome and Metabolome for Health Laboratory, Division of Biotechnology, Faculty of Agro-Industry, Chiang Mai University, Chiang Mai, Thailand
[b]Gut Microbiome Research Group, Mae Fah Luang University, Muang, Chiang Rai, Thailand
[c]Scientific and Technological Instruments Center, Mae Fah Luang University, Chiang Rai, Thailand
[d]School of Science, Mae Fah Luang University, Muang, Chiang Rai, Thailand
[e]Laboratory of Microbial Technology, Division of Applied Molecular Microbiology and Biomass Chemistry, Department of Bioscience and Biotechnology, Faculty of Agriculture, Graduate School, Kyushu University, Fukuoka, Japan

* **Correspondence:** Siam Popluechai, siam@mfu.ac.th

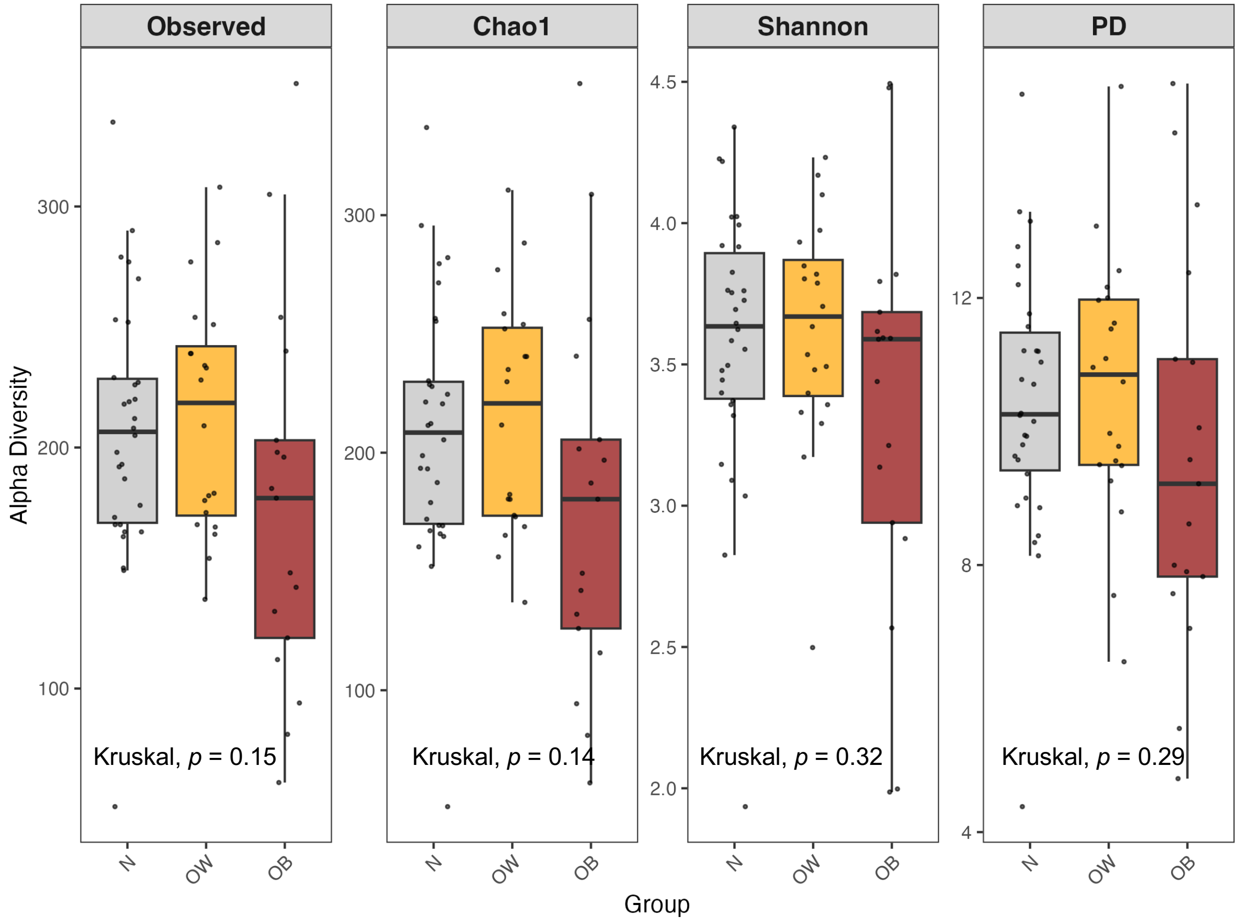

Figure S1 Alpha diversity indices (Observed Species, Chao1 (species richness), Shannon (species richness and evenness), and PD Whole Tree (phylogenetic diversity)) across BMI groups. Microbial diversity was assessed at a sequencing depth of 48,426 reads per sample. Differences in alpha diversity among BMI groups were evaluated using the Kruskal–Wallis test ($p < 0.05$). BMI groups: N = normal weight (n = 30); OW = overweight (n = 20); OB = obese (n = 17).

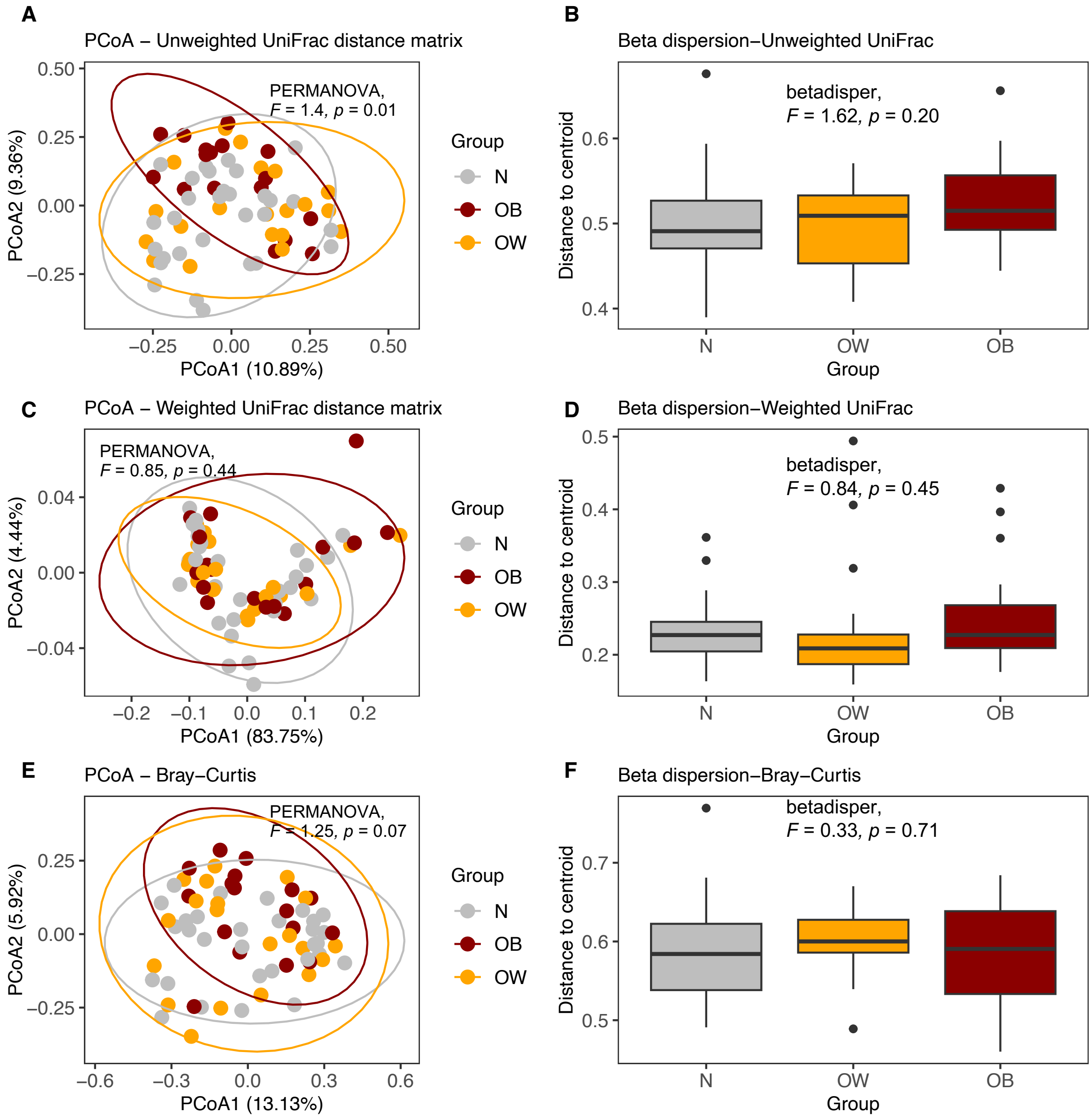

Figure S2 Beta diversity of gut microbiota across BMI groups. (A, C, E) PCoA plots showing gut microbiota composition in children based on unweighted UniFrac, weighted UniFrac, and Bray–Curtis distance matrices, respectively. Differences across BMI groups were assessed using PERMANOVA. (B, D, F) Boxplots illustrating within-group dispersion (distance to centroid) for each BMI group corresponding to the distance matrices. All groups exhibited homogeneous dispersion. BMI groups: N = normal weight (n = 30); OW = overweight (n = 20); OB = obese (n = 17).

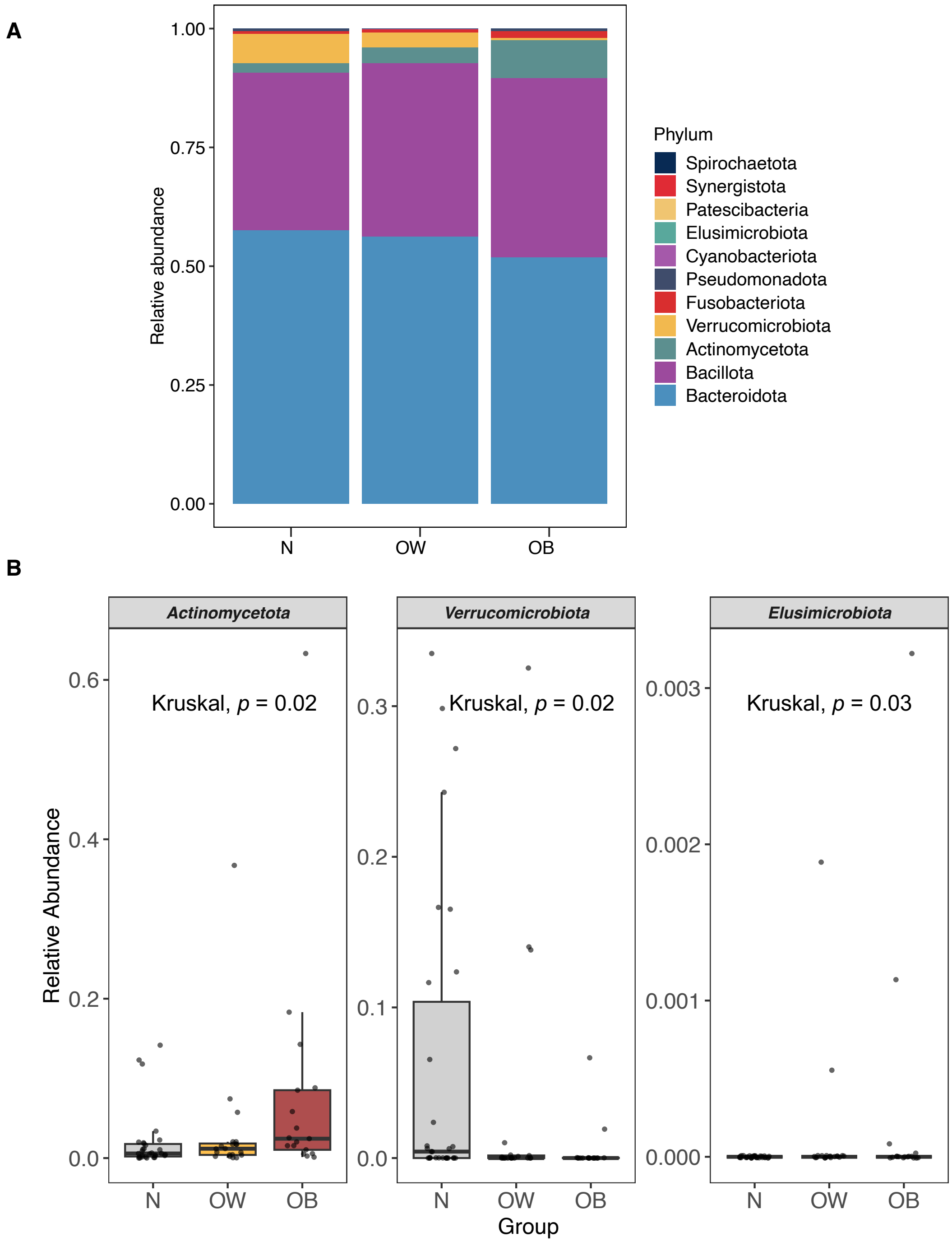

Figure S3 Relative abundance of gut microbiota across BMI groups. (A) Bar plot showing the mean relative abundance of gut microbiota at the phylum level. (B) Box plots displaying significant differences in gut microbiota at the class level among BMI groups. Differences in relative abundance were assessed using the Kruskal–Wallis test ($p < 0.05$), followed by Dunn's post hoc test with Benjamini–Hochberg correction for multiple comparisons. BMI groups: N = normal weight (n = 30); OW = overweight (n = 20); OB = obese (n = 17).

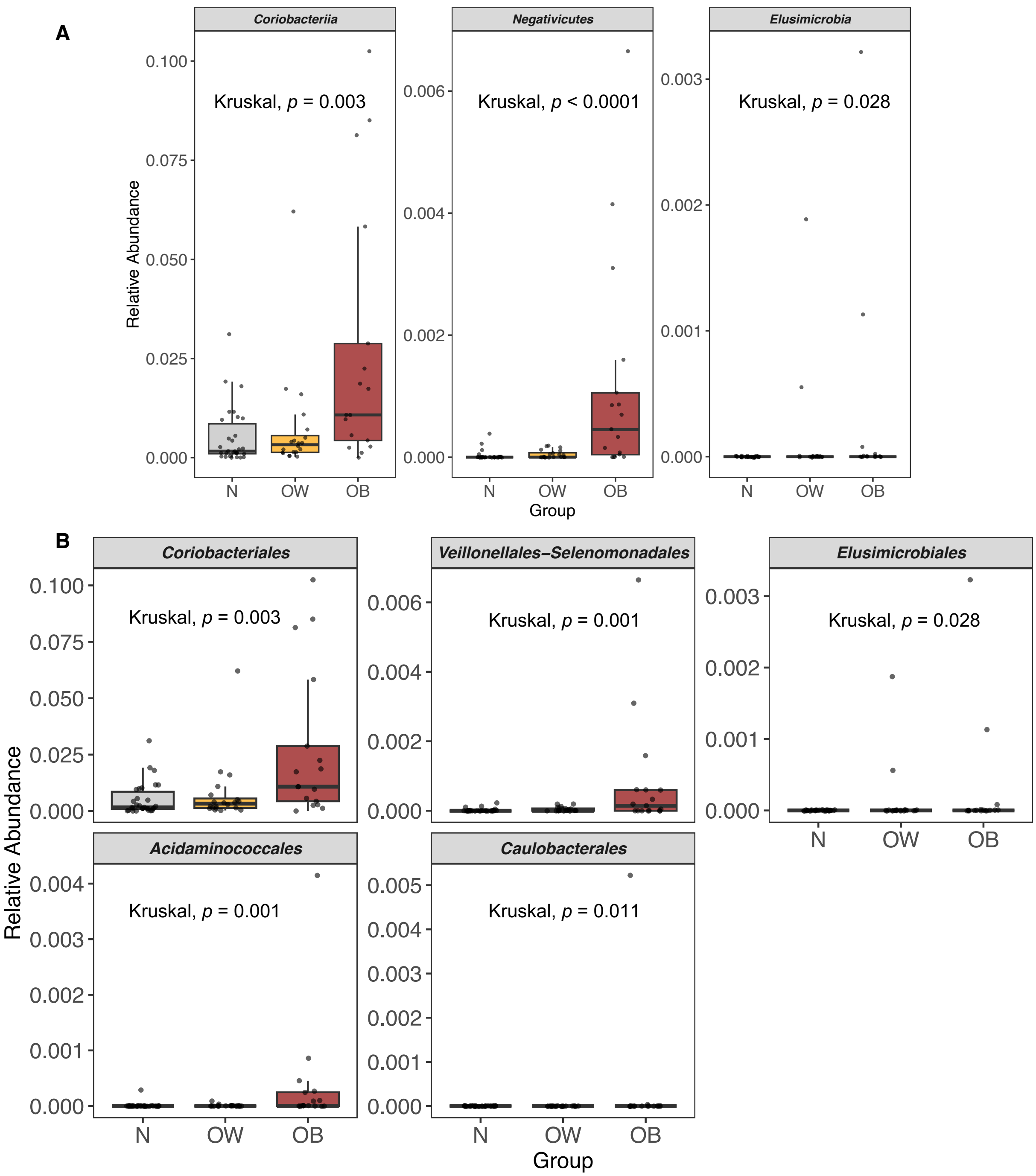

Figure S4 Relative abundance of gut microbiota across BMI groups. (A) Box plots displaying significant differences in gut microbiota at the class level among BMI groups. (B) Box plots displaying significant differences in gut microbiota at the order level among BMI groups. Differences in relative abundance were assessed using the Kruskal–Wallis test ($p < 0.05$), followed by Dunn's post hoc test with Benjamini–Hochberg correction for multiple comparisons. BMI groups: N = normal weight (n = 30); OW = overweight (n = 20); OB = obese (n = 17).

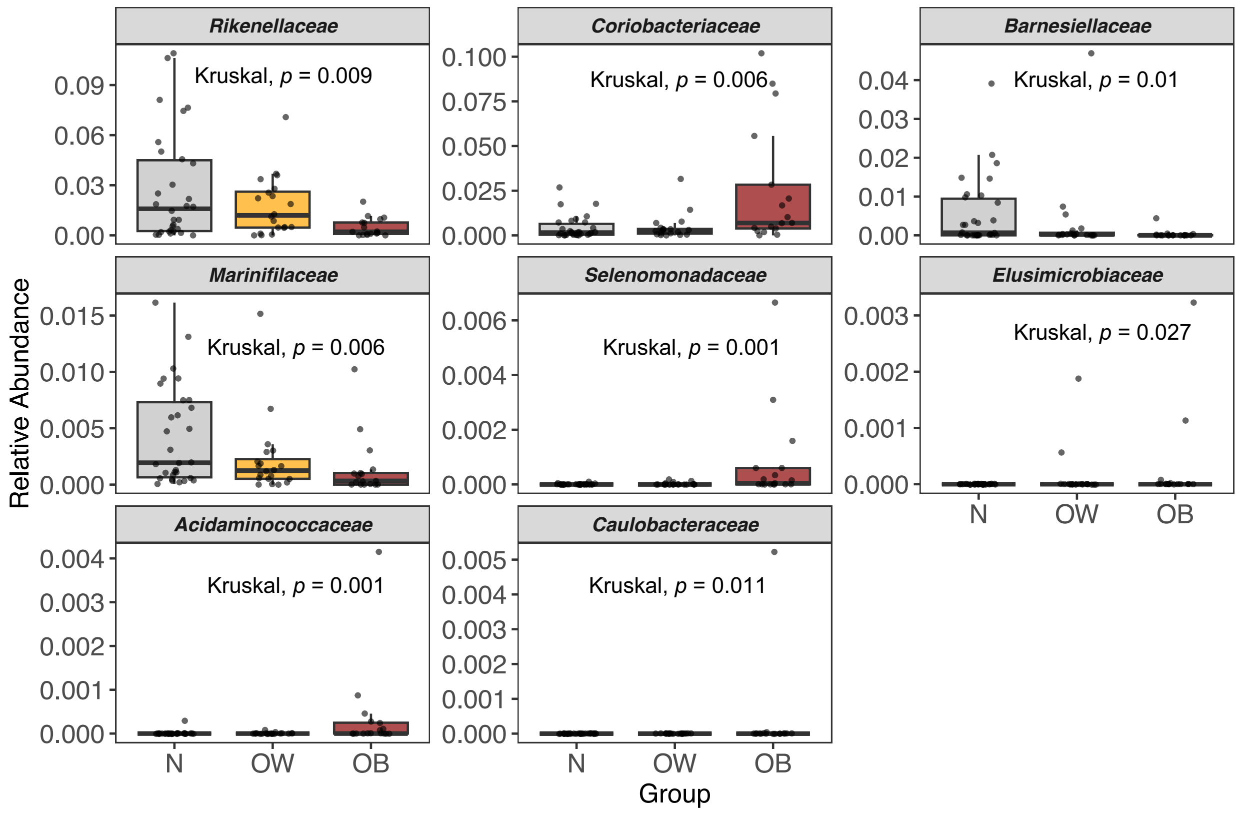

Figure S5 Relative abundance of gut microbiota across BMI groups. Box plots displaying significant differences in gut microbiota at the family level among BMI groups. Differences in relative abundance were assessed using the Kruskal–Wallis test ($p < 0.05$), followed by Dunn's post hoc test with Benjamini–Hochberg correction for multiple comparisons. BMI groups: N = normal weight (n = 30); OW = overweight (n = 20); OB = obese (n = 17).

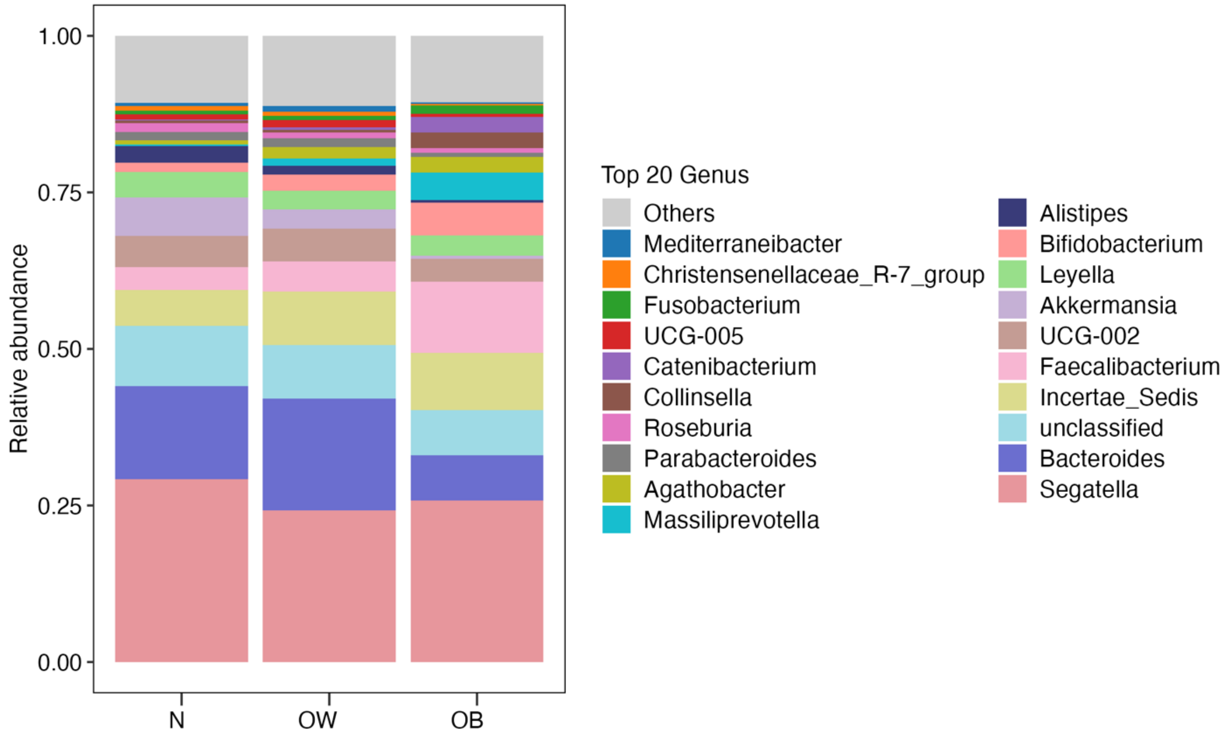

Figure S6 Relative abundance of top 20 genus across BMI groups. Bar plot showing the mean relative abundance of gut microbiota at the genus level. BMI groups: N = normal weight (n = 30); OW = overweight (n = 20); OB = obese (n = 17).

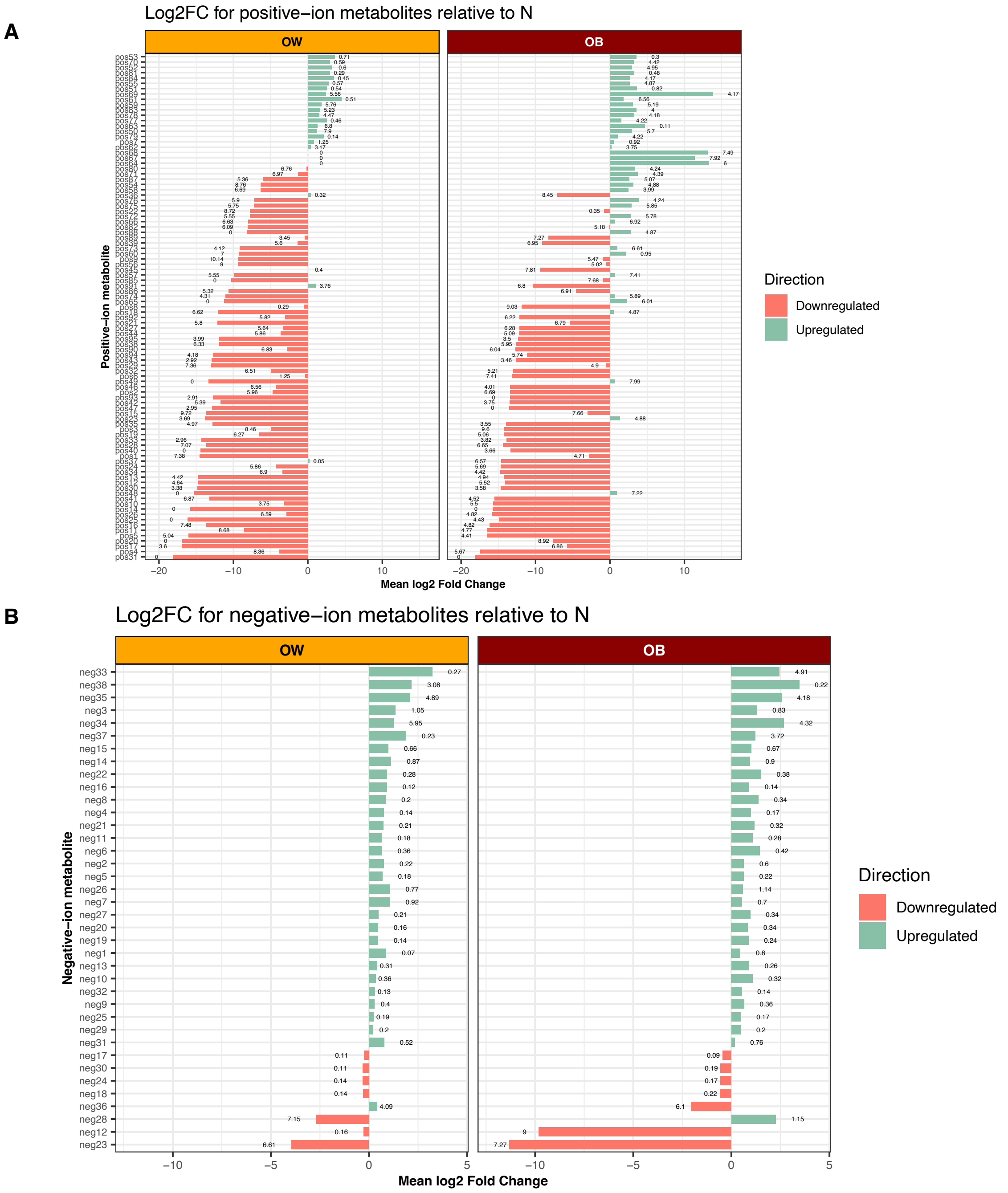

Figure S7 Fecal metabolite abundance across BMI groups. Bar plots of mean Log_2 fold changes (sample-wise) for 95 differential positive-ion (A) and 38 negtive-ion (B) metabolites in the OW and OB groups relative to the N group. Green bars indicate upregulated metabolites, while red bars indicate downregulated metabolites. Bar labels correspond to standard deviation (±SD). Full metabolite names are provided in Supplementary File 4. BMI groups: N = normal weight (n = 24); OW = overweight (n = 18); OB = obese (n = 13).

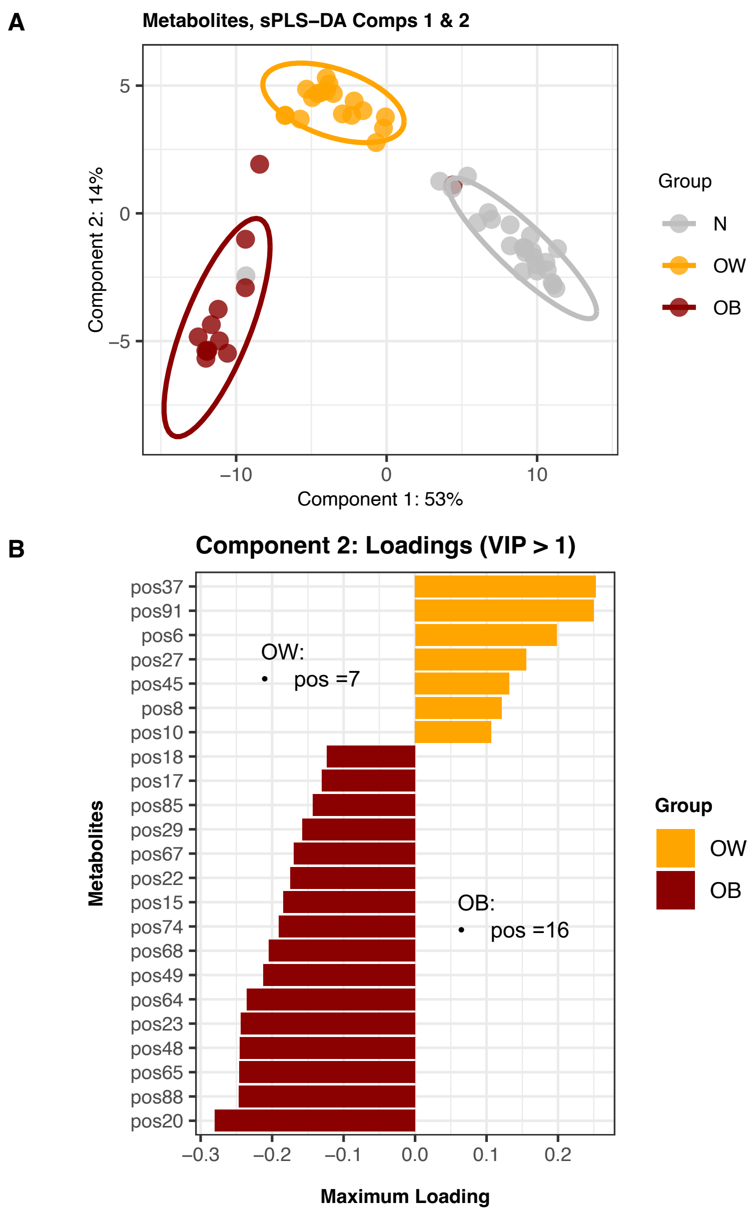

Figure S8 Sparse partial least squares discriminant analysis (sPLS-DA) of fecal metabolites across BMI groups. (A) Individual plot showing sample distribution and grouping based on sPLS-DA components. (B) Bar plots depicting discriminant metabolites identified by the tuned sPLS-DA model (VIP > 1) with the highest loading scores on component 2, based on the maximum (positive) and minimum (negative) group mean coordinates. Horizontal bars represent individual metabolites detected in positive-ion mode, with bar length proportional to their loading weight. BMI groups: N = normal weight (n = 24); OW = overweight (n = 18); OB = obese (n = 13). Abbreviations: pos = positive-ion mode; neg = negative-ion mode.

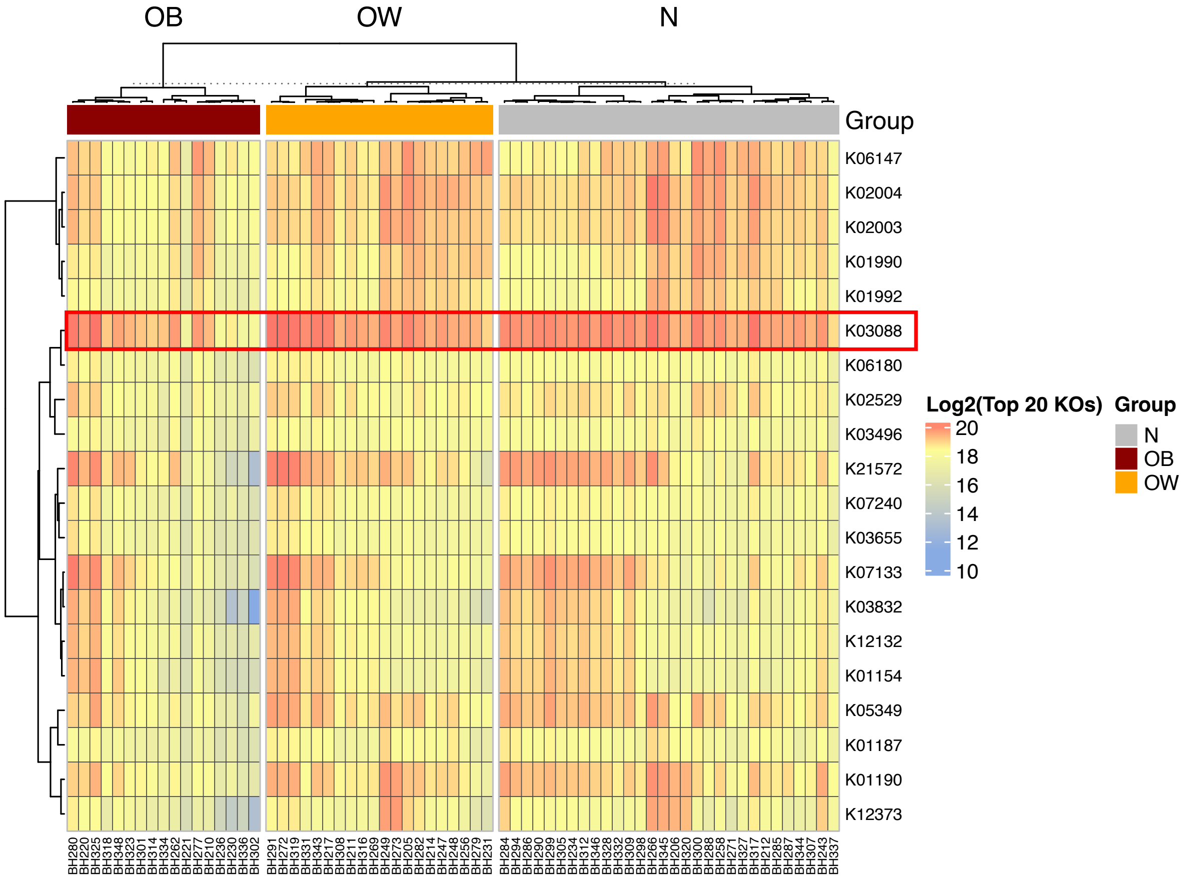

Figure S9. Hierarchical clustering of the top 20 KOs across BMI groups. The heatmap shows the Log_2-transformed abundance of the top 20 KOs, with red indicating higher abundance and blue indicating lower abundance. The red rectangle highlights the most abundant KO (K03088—*rpoE*; RNA polymerase sigma-70 factor, ECF subfamily) across BMI groups. Both rows (KOs) and columns (samples) were clustered using Spearman correlation distance. BMI groups: N = normal weight (n = 30); OW = overweight (n = 20); OB = obese (n = 17). Abbreviations: pos = positive-ion mode; neg = negative-ion mode.

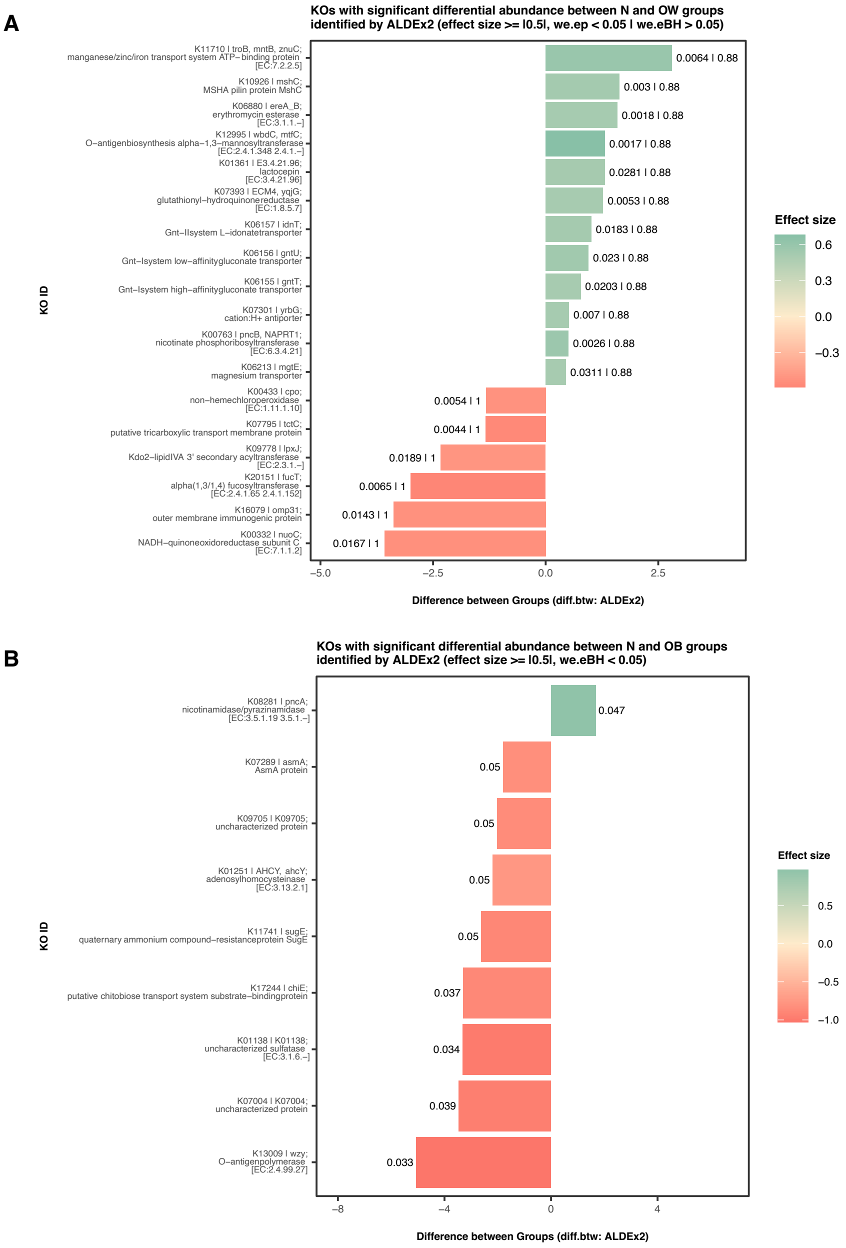

Figure S10 Differentially abundant KEGG Orthologs (KOs) between BMI groups based on ALDEx2 analysis. Bar plot showing significantly differentially abundant KOs between N and OW groups (A; *we.ep* < 0.05, *we.eBH* > 0.05) and between N and OB groups (B; *we.eBH* < 0.05). Green bars (positive differences) indicate higher abundance in the latter group, while red bars (negative differences) indicate lower abundance. Effect size thresholds were defined as follows: ≥ 0.5 (small), 1.0–1.5 (moderate), and > 1.5 (large). A larger effect size indicates a stronger difference between groups relative to within-group variation. Bar labels represent *p*-values (we.ep) and/or Benjamini–Hochberg adjusted *q*-values (*we.eBH*). BMI groups: N = normal weight (n = 30); OW = overweight (n = 20); OB = obese (n = 17).

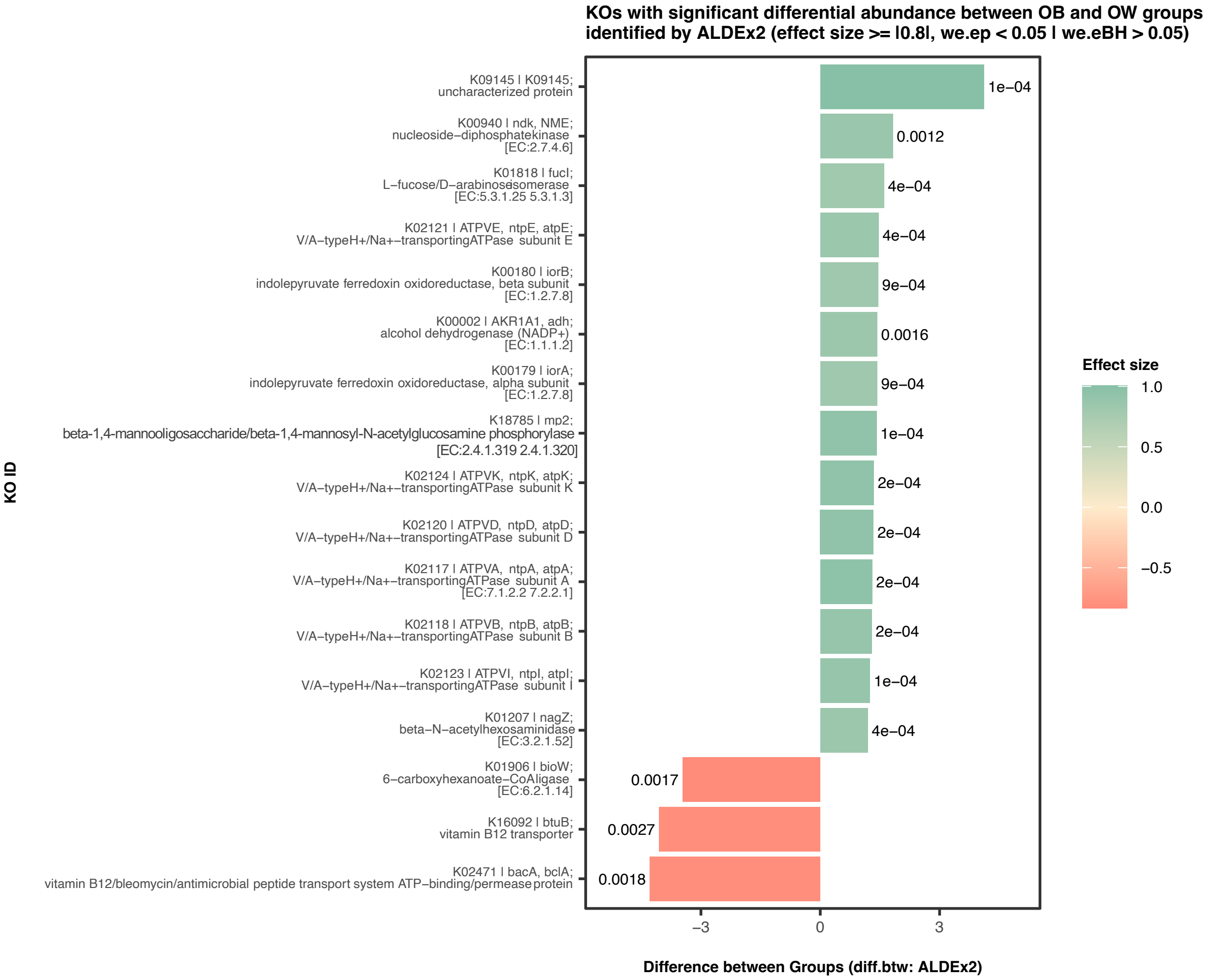

Figure S11 Differentially abundant KEGG Orthologs (KOs) between BMI groups based on ALDEx2 analysis. Bar plot showing significantly differentially abundant KOs between OB and OW groups (*we.ep* < 0.05, *we.eBH* > 0.05). Green bars (positive differences) indicate higher abundance in the latter group, while red bars (negative differences) indicate lower abundance. Effect size thresholds were defined as follows: ≥ 0.5 (small), 1.0–1.5 (moderate), and > 1.5 (large). A larger effect size indicates a stronger difference between groups relative to within-group variation. Bar labels represent *p*-values (we.ep) and/or Benjamini–Hochberg adjusted *q*-values (*we.eBH*). BMI groups: N = normal weight (n = 30); OW = overweight (n = 20); OB = obese (n = 17).

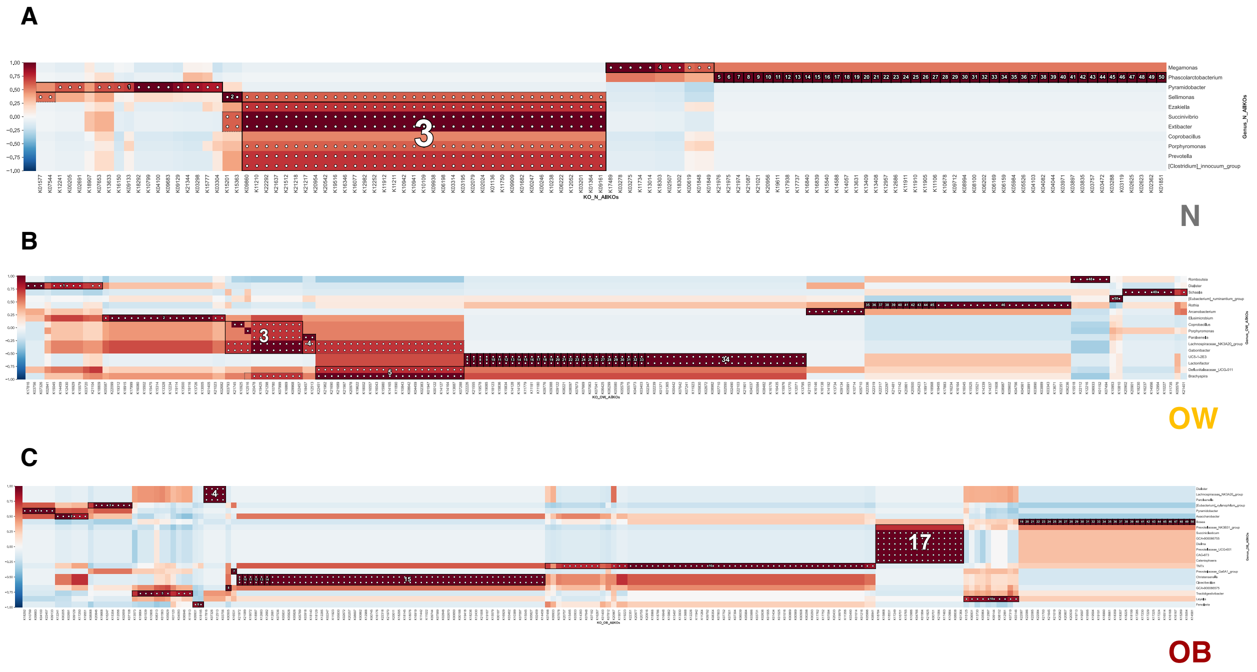

Figure S12 Significant associations between gut microbiota at the genus level and KEGG Orthologs (KOs) based on HAllA analysis. Heatmaps display significant correlations ($q < 0.05$) between gut microbiota and KOs in the (A) N, (B) OW, and (C) OB groups. Red indicates a positive association, while blue indicates a negative association. The analysis includes 144 genera and 5,819 predicted KOs. BMI groups: N = normal weight (n = 30); OW = overweight (n = 20); OB = obese (n = 17).

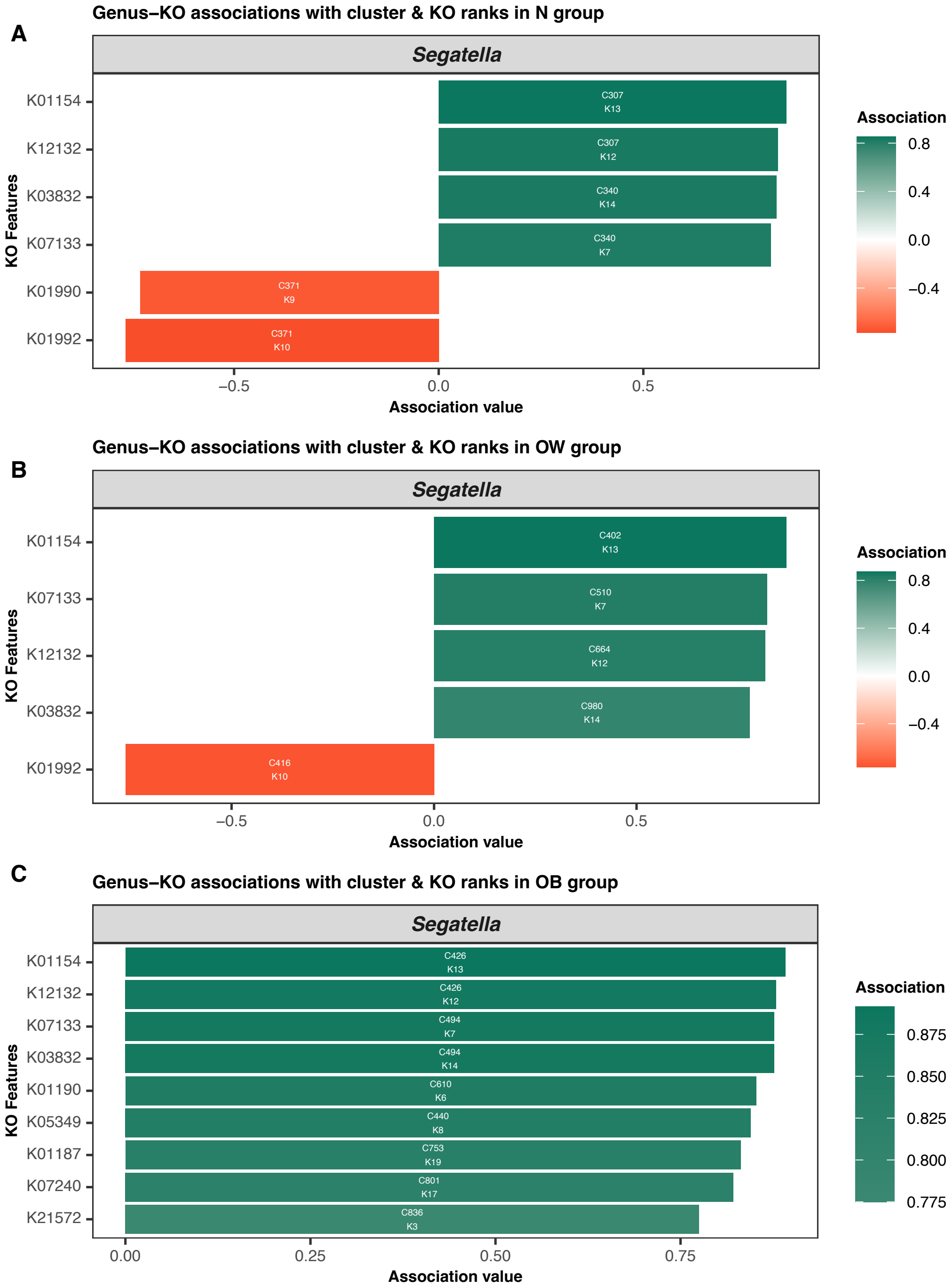

Figure S13 Significant associations between gut microbiota at the genus level and KEGG Orthologs (KOs) based on HAllA analysis. Bar plot showing significant associations ($q < 0.05$) between *Segatella* and the top 20 most abundant KOs in the (A) N, (B) OW, and (C) OB groups. Red bars indicate negative associations; green bars indicate positive associations. Bar labels represent the HAllA cluster rank ("C") and KO rank ("K") BMI groups: N = normal weight (n = 30); OW = overweight (n = 20); OB = obese (n = 17).

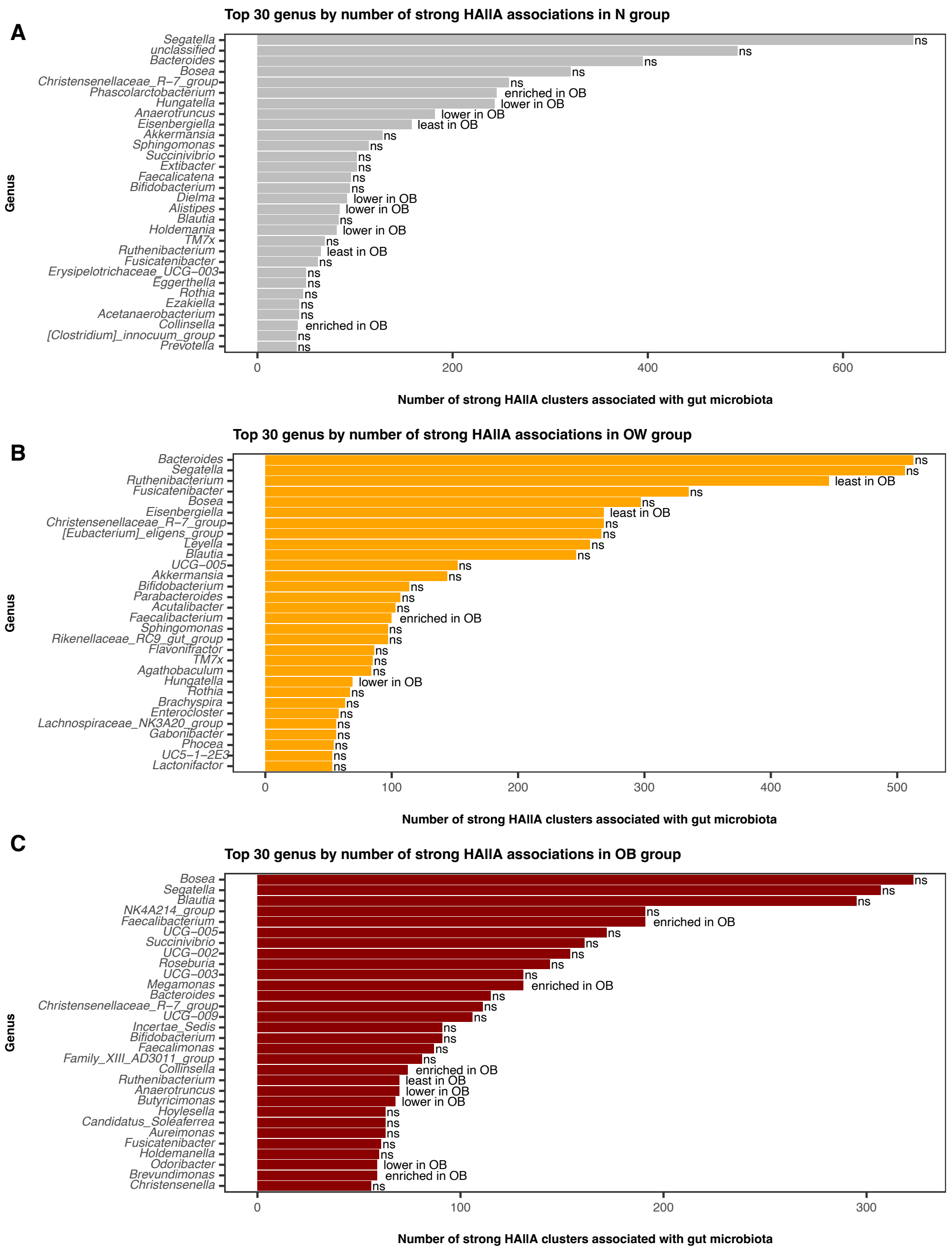

Figure S14. Enrichment of associations between gut microbiota (genus level) and KEGG Orthologs (KOs) across BMI groups based on HAllA analysis. Bar plots show the number of strong HAllA clusters ($r \geq |0.7|$, $q < 0.05$) associated with gut microbial genera in (A) normal weight (N; grey bars), (B) overweight (OW; yellow bars), and (C) obese (OB; dark red bars) groups. Bar labels indicate genera that were significantly different in abundance between BMI groups, identified by post hoc Dunn's test with Benjamini-Hochberg correction ($q < 0.05$), following a significant Kruskal–Wallis test ($p < 0.05$). BMI groups: N = normal weight (n = 30); OW = overweight (n = 20); OB = obese (n = 17).

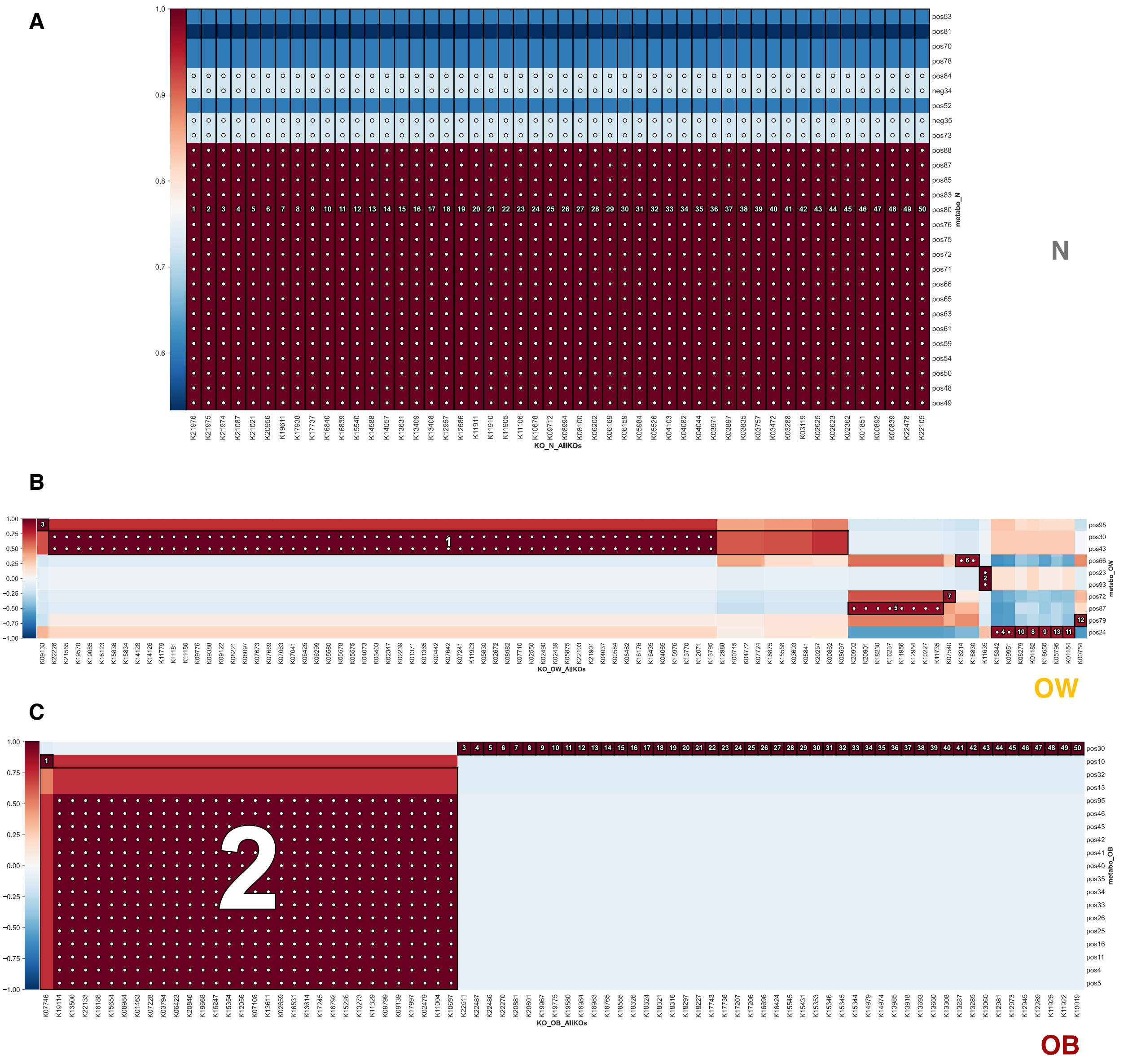

Figure S15 Significant associations between metabolites and KEGG Orthologs (KOs) based on HAllA analysis. Heatmaps display significant correlations ($q < 0.05$) between metabolites and KOs in the (A) N, (B) OW, and (C) OB groups. Red indicates a positive association, while blue indicates a negative association. The analysis includes 133 metabolites (95 detected in positive-ion mode and 38 in negative-ion mode) and 5,819 predicted KOs. BMI groups: N = normal weight (n = 24); OW = overweight (n = 18); OB = obese (n = 13).

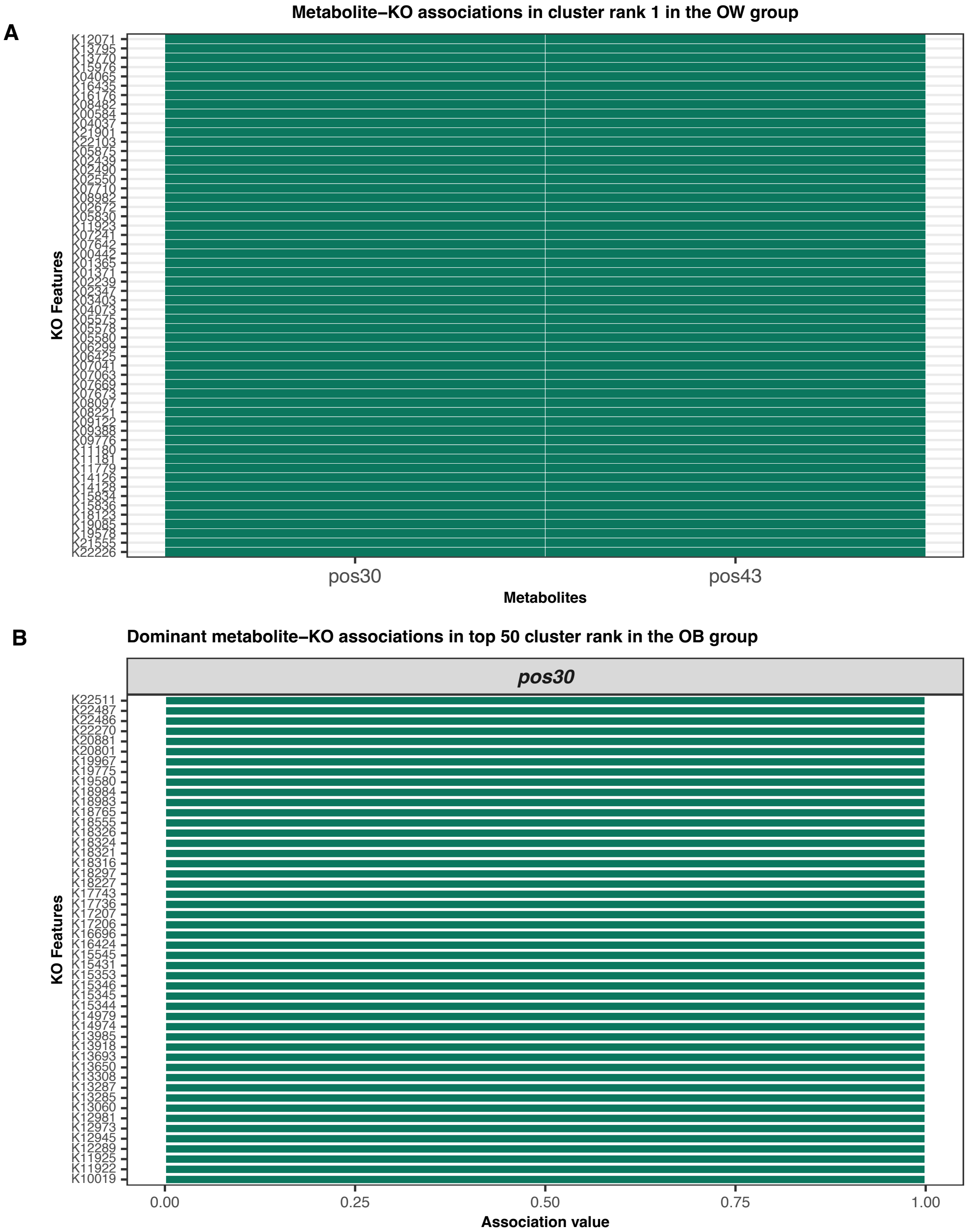

Figure S16. Significant associations between dominant metabolites and KEGG Orthologs (KOs) based on HAllA analysis. (A) Heatmaps showing significant correlations ($q < 0.0001$) between dominant metabolites and KOs in cluster rank 1 in the OW group. (B) Bar plot showing significant associations ($q < 0.0001$) between a dominant metabolite and KOs within the top 50 cluster ranks in the OB group. Green bars indicate very strong associations ($r = 1$, $q < 0.0001$). BMI groups: N = normal weight (n = 24); OW = overweight (n = 18); OB = obese (n = 13).

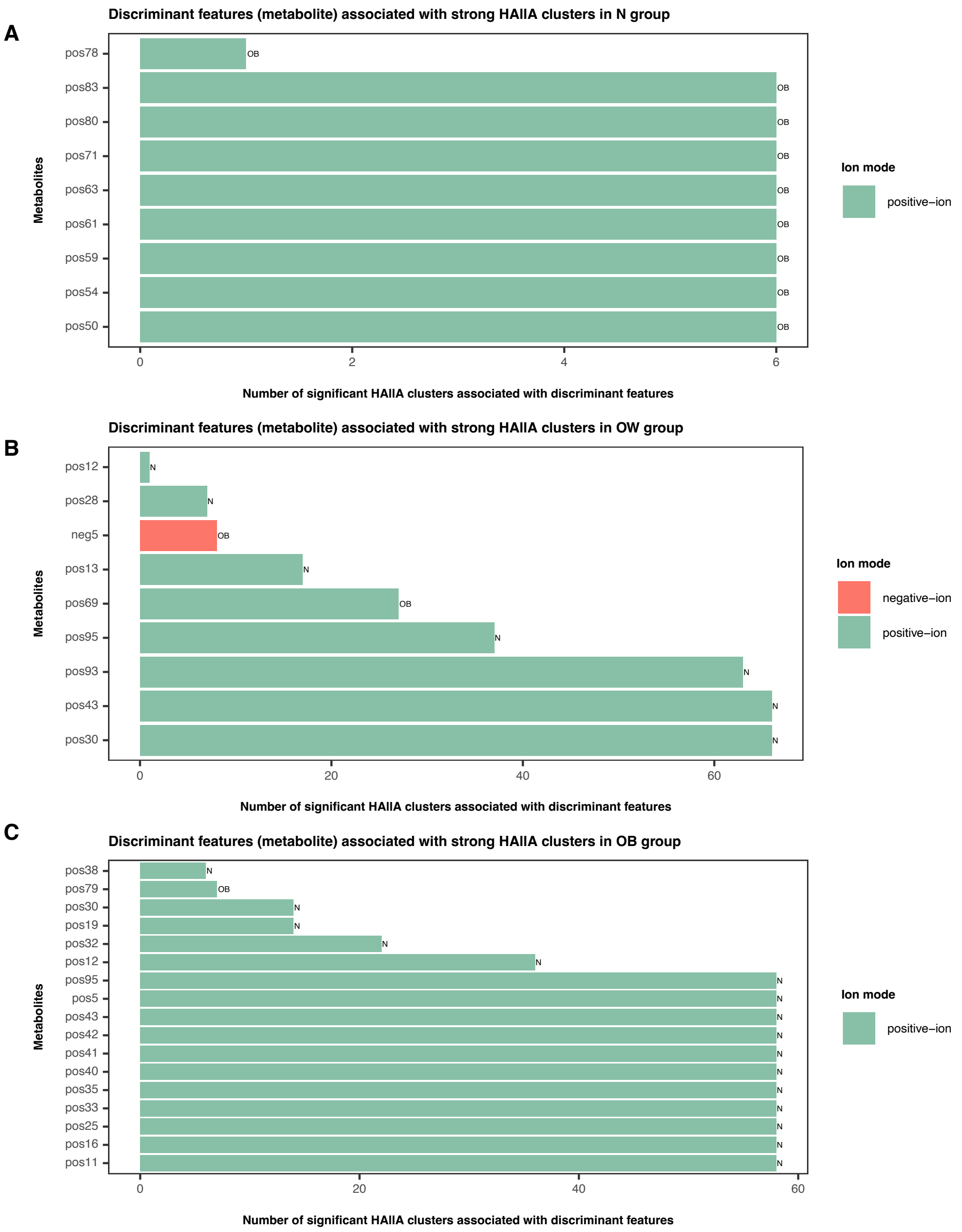

Figure S17 Discriminant metabolite features associated with strong HAllA clusters (integrating metabolites and KO|Genus) in each BMI group. Bar plots illustrate the number of significant HAllA clusters linked to discriminant metabolite features identified by the sPLS-DA tuned model (VIP > 1) in (A) normal weight (N), (B) overweight (OW), and (C) obese (OB) groups. Green bars represent metabolites detected in positive-ion mode, while red bars represent those detected in negative-ion mode. Bar labels correspond to discriminant metabolites contributing to BMI group separation (VIP > 1) as determined by the sPLS-DA model. Full compound names are listed in Supplementary File 4. BMI groups: N = normal weight (n = 24); OW = overweight (n = 18); OB = obese (n = 13).
