# Supplementary material for: Gut microbiome and fecal metabolite profiles in obese school-aged children from Northern Thailand
Source: Front Microbiol. 2025 Sep 11;16:1657839. doi: 10.3389/fmicb.2025.1657839 (PMC12461226; doi:10.3389/fmicb.2025.1657839)
Supplement: Supplementary file 4 [file Data_Sheet_4.PDF]

Gut Microbiome and Fecal Metabolite Profiles in Obese School-Aged Children from Northern Thailand

Phatthanaphong Therdtatha[a], Lucsame Gruneck[b], Poramet Nachalam[b, c], Vasana Jinatham[b, d], Kritsakorn Saninjuk[b, d], Jiro Nakayama[e], Siam Popluechai[b, d*]

[a]Specialized Research in Microbiome and Metabolome for Health Laboratory, Division of Biotechnology, Faculty of Agro-Industry, Chiang Mai University, Chiang Mai, Thailand
[b]Gut Microbiome Research Group, Mae Fah Luang University, Muang, Chiang Rai, Thailand
[c]Scientific and Technological Instruments Center, Mae Fah Luang University, Chiang Rai, Thailand
[d]School of Science, Mae Fah Luang University, Muang, Chiang Rai, Thailand
[e]Laboratory of Microbial Technology, Division of Applied Molecular Microbiology and Biomass Chemistry, Department of Bioscience and Biotechnology, Faculty of Agriculture, Graduate School, Kyushu University, Fukuoka, Japan

* **Correspondence:** Siam Popluechai, siam@mfu.ac.th

Table S1. Major contributing metabolites to variation on Dimension 1 (MFA).

Associated group	Code	Metabolite	correlation with Dim 1	*p*.value
N	neg17	1-[2-Fluoro-4-(methanesulfonyl) phenyl]-1,4-diazepane	$r = -0.90$	< 0.0001
N	neg18	Hoechst 33342	$r = -0.91$	< 0.0001
N	neg30	3'?-Isopravastatin	$r = -0.89$	< 0.0001
N	neg24	Asn His Val	$r = 0.88$	< 0.0001
OB	neg8	Sarcostin	r = 0.95	< 0.0001
OB	neg22	Phe Glu Arg	$r = 0.95$	< 0.0001
OB	neg21	15-keto-Prostaglandin E2	$r = 0.94$	< 0.0001
OB	neg11	JWH 018 N-(5-hydroxypentyl) metabolite-d5	$r = 0.94$	< 0.0001
OB	neg4	Ala Lys Pro Gln	$r = 0.94$	< 0.0001
OB	neg6	Imidiocarb	$r = 0.93$	< 0.0001
OB	neg19	L-Isoleucyl-L-tyrosyl-L-leucyl-L-histidine	$r = 0.93$	< 0.0001

Table S2. Gut microbiota strongly associated with positive-ion metabolites in the OB group, identified by RDA scaling 2.

Genus	Code	Metabolite
Romboutsia, *Collinsella*	pos87	Glycyl-L-prolyl-L-tyrosyl-L-alanyl-L-prolyl-L-alanylglycyl-L-histidine
	pos75	Thalicarpine
	pos76	Nimbolidin C
	pos69	unclassified
Megamonas	pos68	unclassified
	pos67	unclassified
	pos64	unclassified
	pos88	Amaranthussaponin II
	pos65	PC(16:0/9:0(CHO))
	pos48	unclassified
	pos49	unclassified

Table S3. Metabolites with the highest number of KO associations identified by HAllA in each BMI group.

Group	Code	Metabolite	Number of KO associations
N	neg34	Granisetron metabolite 4 glucuronide	322
N	pos84	1-Piperidinebutanol, alpha,alpha-bis((3,4-(methylenedioxy)phenoxy) methyl)-, methylcarbamate (ester), citrate	322
OW	pos30	9-Fluoro-16alpha-hydroxyandrost-4-ene-3,11,17-tri ne	56
OW	pos43	Isoferulic acid	56
OB	pos30	9-Fluoro-16alpha-hydroxyandrost-4-ene-3,11,17-trione	169
